# Non-linear associations between parity and long-term risk of osteoporosis

**DOI:** 10.1007/s11657-026-01766-x

**Published:** 2026-11-05

**Authors:** Wanqiong Yuan, Jiaojiao Liao, Liyuan Tao, Haoze Song, Huan Wang, Jie Huang, Jinwen He, Hao Liu, Tong Wu, Hao Zhou, Yundi Zhang, Chunli Song

**Affiliations:** 1 Department of Orthopedics, Peking University Third Hospital, Beijing, 100191 China; 2 Engineering Research Center of Bone and Joint Precision Medicine, Ministry of Education, Beijing, 100191 China; 3 Beijing Key Laboratory of Advanced Bioadaptable Orthopedic Implants, Beijing, 100191 China; 4 Research Center of Clinical Epidemiology, Peking University Third Hospital, Beijing, 100191 China; 5 Medical Examination Centre, Peking University Third Hospital, Beijing, 100191 China; 6 Beijing Normal University, School of Mathematical Science, Beijing, 100875 China

**Keywords:** Parity, Osteoporosis, Osteoporotic fracture, Body mass index, Age, UK Biobank

## Abstract

***Summary*:**

Our findings suggest that although pregnancy and lactation are periods of physiological calcium loss, higher parity is not associated with an increased risk of postmenopausal osteoporosis; rather, it appears to confer a long-term protective effect.

**Purpose:**

Osteoporosis is a significant public health burden. While parity induces transient bone loss, their long-term skeletal impact remains poorly understood. This study aimed to clarify the associations between parity, defined as the number of live births, and the risk of osteoporosis, osteoporotic fractures, and bone mineral density (BMD).

**Methods:**

We analyzed data from 119,969 women in the UK Biobank. Associations between parity and incident osteoporosis or fractures were evaluated using unweighted and inverse probability of treatment weighting Cox proportional hazards models. Generalized linear models assessed associations with femoral neck and lumbar spine BMD. Models were adjusted for sociodemographic, genetic, lifestyle, and clinical factors.

**Results:**

Compared with nulliparous women, those with a parity of 2 or 3 exhibited a significantly reduced risk of osteoporosis in the weighted fully adjusted model. In addition, parity of 3 exhibited a significantly reduced risk of osteoporotic fracture compared with the nulliparous group. This protection was evident in women ≥ 60 years and those with normal body mass index. Parity was not significantly associated with areal BMD changes after full covariate adjustment.

**Conclusion:**

In women aged 40–69 years at baseline, a parity of 2 or 3 confers long-term protection against osteoporosis and osteoporotic fracture (a arity of 3), occurring independently of BMD changes. These findings support the integration of parity into clinical risk assessment tools, ultimately contributing to more effective osteoporosis prevention and a reduced fracture burden across women’s lifespan.

**Supplementary Information:**

The online version contains supplementary material available at https://doi.org/10.1007/s11657-026-01766-x.

## Introduction

Osteoporosis poses a major public health challenge, primarily characterized by osteoporotic fractures, which lead to substantial disability, mortality, and socioeconomic burdens [1–3]. Women face the highest risk for developing osteoporosis and sustaining related fractures, highlighting the critical importance of maintaining bone health throughout the lifespan of women [4]. Although parity, which is the condition of having borne children, significantly influences bone health [5–8], its long-term skeletal legacy remains poorly characterized. Clarifying this relationship is essential for developing effective preventive strategies against osteoporosis in women.

The maternal bone undergoes profound metabolic adaptations during the childbearing cycle, marked by significantly accelerated bone turnover [9, 10]. To meet the demands of fetal bone mineralization during pregnancy and lactation, mothers undergo substantial calcium loss and a state of accelerated bone turnover, leading to a transient decline in bone mineral density and an elevated risk of osteoporosis and osteoporotic fractures. This phenomenon has traditionally positioned parity as a potential risk factor for long-term skeletal health in women [11–13]. Although rare and acute skeletal events can manifest during reproductive intervals in the form of pregnancy- and lactation-associated osteoporosis (PLO) [14], such localized risk profiles are distinct from the long-term, postmenopausal skeletal longevity that spans across the female lifespan.

Several studies indicate that multiparity is associated with unchanged or even reduced risk of osteoporosis and osteoporotic fracture [10, 15–18]. This observation reveals a knowledge gap between short-term physiological depletion and long-term bone health. It has been reported that parity induces trabecular bone loss and simultaneously optimizes the structure and mechanical properties of cortical bone, implying a potential protective role of higher parity against osteoporosis [19–22].

In this prospective cohort study, we systematically evaluated the associations of parity with three primary endpoints: osteoporosis, osteoporotic fractures and bone mineral density (BMD) using the UK Biobank. To further elucidate these relationships, we conducted subgroup analyses stratified by age and body mass index (BMI). We aimed to clarify the complex relationship between parity and long-term skeletal health, providing evidence-based guidance for developing personalized prevention strategies and lifelong health management approaches for women. Ultimately, implementing bone health initiatives that account for parity could substantially reduce the future burden of osteoporosis and osteoporotic fractures.

## Methods

### Study design and population

We used data from the UK Biobank, a multicenter, prospective, population-based study with 502,236 participants aged 40 to 69 years old identified from National Health Service primary care registers and enrolled at 22 assessment sites across England, Scotland, and Wales between 2006 and 2010. The study received ethical approval from the Northwest Multi-center Research Ethics Committee (reference 21/NW/0157). At the baseline assessment visit, they completed a computer-assisted interview and a touch-screen questionnaire on sociodemographic, lifestyle, and clinical characteristics, provided biological samples, and underwent physical and medical examinations. Disease diagnoses were subsequently followed up through linkage with electronic health records and national registries. Mortality outcomes were ascertained via linkage to national death registries. Detailed study protocols have been published elsewhere [23].

**Fig. 1:**
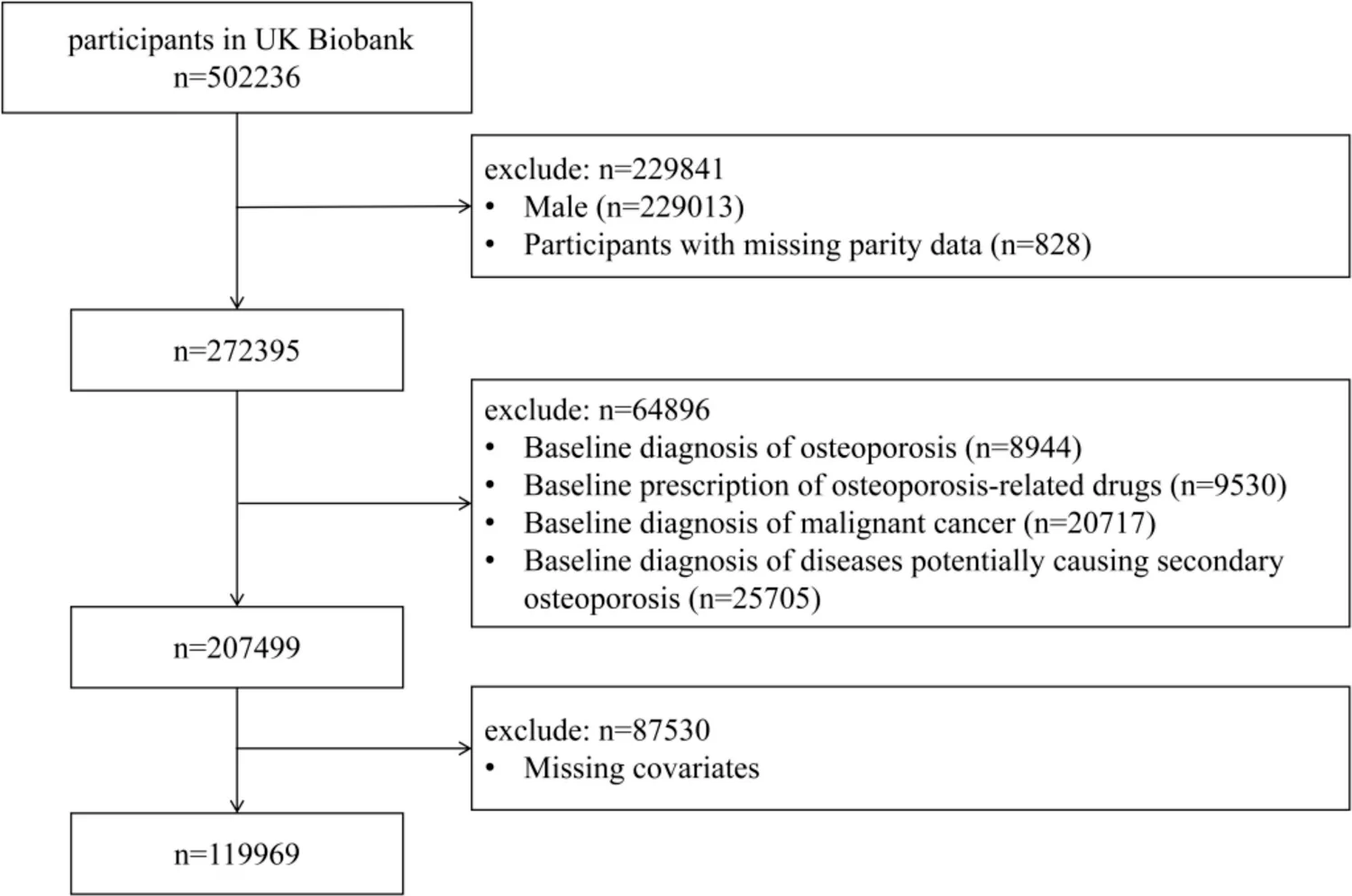
Study flowchart. A schematic illustrating the inclusion and exclusion process used to assess the roles of parity in bone

Participants with missing data on parity or covariates were excluded, as well as those with a history of malignant cancer, hyperthyroidism, hypothyroidism, osteoarthritis, or inflammatory bowel disease at baseline, those with prior prescriptions for osteoporosis-related medications, or those already diagnosed with osteoporosis at baseline. After exclusions, 119,969 participants were included in the final analysis. The participant selection process was illustrated in Fig. 1. This study followed the Strengthening the Reporting of Observational Studies in Epidemiology (STROBE) reporting guideline.

### Assessment of parity, osteoporosis and bone density

At baseline, the number of live births was obtained from the self-reported responses from participants to the touchscreen question: “How many children have you given birth to? (Please include live births only).” The corresponding UK Biobank field ID is listed in Supplementary Table 1. For analyses with incident osteoporosis as the primary outcome, participants were followed from baseline until the earliest occurrence of (1) first diagnosis of osteoporosis (ICD-10: M80–M82), (2) death, or (3) end of follow-up (November 30, 2022).

For analyses with osteoporotic fracture as the outcome, follow-up continued until the first diagnosis of an osteoporotic fracture, death, or the end of follow-up, whichever occurred first. Bone mineral density (BMD) at the neck of the femur and lumbar spine was measured using a dual-energy X-ray absorptiometry (DXA) device (iDXA, GE Lunar, Madison, WI). DXA measurements were conducted from 2014 onwards.

### Measurements of covariates

Potential covariates were selected based on prior evidence and biological relevance and included sociodemographic, genetic, lifestyle, socioeconomic, anthropometric, and clinical factors: age (continuous), BMI (normal, underweight, overweight, obese), ethnicity (White, Asian, Black, Mixed, Other), education (college or university, not), standard polygenic risk score for osteoporosis (continuous), smoking (never, occasionally, daily or almost daily), drinking (never/special occasions only, 1–3 times/month, 1–2 times/week, 3–4 times/week, daily/almost daily), diet (healthy, unhealthy), physical activity (low, moderate, high; assessed by the International Physical Activity Questionnaire, IPAQ), residential area (urban, town, village), employment status (unemployed, full- or part-time student, doing unpaid or voluntary work, retired, in paid employment or self-employed), Townsend deprivation index (continuous), grip strength (weak, not weak), age at menarche (≤ 11, 12–15, ≥ 16 years), and menopausal status (yes, no). Clinical comorbidities at baseline included coronary heart disease (CHD), hypertension, chronic kidney disease (CKD), type 1 diabetes mellitus (T1DM), type 2 diabetes mellitus (T2DM), heart failure (HF), stroke, and atherosclerosis (AS), all identified from hospital inpatient records (UKB field ID 41270). Detailed field IDs for all variables are provided in Supplementary Table 1.

BMI was calculated as weight (kg)/height (m^2^) and categorized as underweight (< 18.5), normal (18.5–24.9), overweight (25.0–29.9), or obese (≥ 30). A healthy diet was defined according to standard UK Biobank dietary criteria.

Grip strength was measured using a Jamar hydraulic hand dynamometer (Lafayette Instrument Company, Lafayette, IN), with weakness defined as maximum strength < 18.0 kg in both hands [24]. Menarche age and menopausal status were self-reported at baseline.

### Statistical analysis

We first examined the association between parity and the risk of osteoporosis and osteoporotic fracture using Cox proportional hazards models. To reduce confounding and achieve better balance in baseline characteristics among different parity groups, we applied inverse probability of treatment weighting (IPTW) to construct a weighted pseudo-population. The propensity score was estimated using a logistic regression model including the following covariates: age, BMI, ethnicity, education, smoking, drinking, diet, IPAQ physical activity level, residential area, employment, Townsend deprivation index, age at menarche, polygenic risk score for osteoporosis, and grip strength. Weighted Cox models were subsequently fitted to estimate hazard ratios (HRs) and 95% confidence intervals (CIs) for the associations of parity with osteoporosis and osteoporotic fracture risks. Associations between parity and BMD at the femoral neck and lumbar spine were examined using generalized linear models (GLMs).

Subgroup analyses were performed stratified by age and BMI to evaluate potential effect modification in the associations between parity and osteoporosis. Sensitivity analyses excluded participants diagnosed with osteoporosis or fracture within the first two years of follow-up to minimize reverse causation. All analyses were conducted using R software, version 4.2.2. Two-sided p < 0.05 was considered statistically significant.

## Results

### Study population and baseline characteristics

The flow of participant selection is detailed in Fig. 1. After applying the inclusion and exclusion criteria, we included 119,969 women in the final cohort in the final study cohort.

During follow-up, 5,390 participants (4.5%) were diagnosed with osteoporosis (Table 1). Participants with osteoporosis were significantly older (mean age: 60.45 ± 6.32 years vs. 54.67 ± 8.16 years, *P* < 0.001) and had a shorter mean reproductive duration compared to the non-osteoporosis group (8.27 ± 3.75 years vs. 13.61 ± 1.50 years, *P* < 0.001). A higher proportion of the osteoporosis group had reached menopause (91.6% vs. 64.2%, *P* < 0.001). Osteoporotic participants also exhibited a lower mean BMI (25.55 ± 4.61 vs. 26.63 ± 4.95, *P* < 0.001), with a greater percentage falling into the normal weight (50.5% vs. 42.6%) and underweight (1.8% vs. 0.7%) categories. In terms of clinical and lifestyle factors, the osteoporosis group showed a higher prevalence of weak grip strength (12.5% vs. 6.6%, *P* < 0.001) and a greater likelihood of having ever used hormone therapy (47.2% vs. 29.4%, *P* < 0.001). They also had a significantly higher burden of cardiovascular-related comorbidities, including CHD (3.9% vs. 1.8%, P < 0.001), hypertension (25.4% vs. 19.0%, *P* < 0.001), and stroke (0.4% vs. 0.1%, *P* < 0.001). Weighted baseline characteristics among the parity groups showed balanced covariate distribution after IPTW (Supplementary Table 2–3, Supplementary Fig. 1).

**Table 1:** Baseline characteristics of participants grouped by osteoporosis

	Non-Osteoporosis	Osteoporosis	*P*
n	114,579	5,390	
Duration (years, mean (SD))	13.61 (1.50)	8.27 (3.75)	< 0.001
Age	54.67 (8.16)	60.45 (6.32)	< 0.001
Age ≥ 60 (%)	39,223 (34.2)	3,416 (63.4)	< 0.001
BMI (mean (SD))	26.63 (4.95)	25.55 (4.61)	< 0.001
BMI (%)			< 0.001
Normal	48,866 (42.6)	2,724 (50.5)	
Underweight	802 (0.7)	95 (1.8)	
Overweight	41,468 (36.2)	1,811 (33.6)	
Obesity	23,443 (20.5)	760 (14.1)	
Ethnicity (%)			< 0.001
White	108,736 (94.9)	5,240 (97.2)	
Asian	2,109 (1.8)	65 (1.2)	
Black	1,882 (1.6)	29 (0.5)	
Mixed	852 (0.7)	19 (0.4)	
Other	1,000 (0.9)	37 (0.7)	
College or University	43,054 (37.6)	1,755 (32.6)	< 0.001
Smoking (%)			< 0.001
Never	69,495 (60.7)	3,111 (57.7)	
Occasionally	15,639 (13.6)	733 (13.6)	
Daily or almost daily	29,445 (25.7)	1,546 (28.7)	
Drinking (%)			
Never	8,580 (7.5)	513 (9.5)	< 0.001
Special occasions only	14,733 (12.9)	809 (15.0)	
One to three times a month	14,761 (12.9)	614 (11.4)	
Once or twice a week	30,404 (26.5)	1,261 (23.4)	
Three or four times a week	26,084 (22.8)	1,126 (20.9)	
Daily or almost daily	20,017 (17.5)	1,067 (19.8)	
Healthy diet (%)	72,791 (63.5)	3,546 (65.8)	0.001
IPAQ (%)			0.446
Low	19,745 (17.2)	964 (17.9)	
Moderate	49,208 (42.9)	2,287 (42.4)	
High	45,626 (39.8)	2,139 (39.7)	
Area (%)			0.312
Urban	97,973 (85.5)	4,634 (86.0)	
Town	7,886 (6.9)	376 (7.0)	
Village	8,720 (7.6)	380 (7.1)	
Employment (%)			< 0.001
Unemployed	9,175 (8.0)	356 (6.6)	
Full- or part-time student	71,696 (62.6)	2,182 (40.5)	
Doing unpaid or voluntary work	728 (0.6)	26 (0.5)	
Retired	475 (0.4)	10 (0.2)	
In paid employment or self-employed	32,505 (28.4)	2,816 (52.2)	
Townsend index	−1.47 (2.96)	−1.44 (2.99)	0.457
Menarche age (%)			< 0.001
≤ 11	21,554 (18.8)	924 (17.1)	
12–15	86,549 (75.5)	4,097 (76.0)	
≥ 16	6,476 (5.7)	369 (6.8)	
Standard PRS (mean (SD))	−0.07 (0.93)	0.26 (0.90)	< 0.001
Weak grip strength (%)	7,598 (6.6)	673 (12.5)	< 0.001
Ever hysterectomy (%)	6,700 (5.8)	546 (10.1)	< 0.001
Ever bilateral oophorectomy (%)	3,086 (2.7)	261 (4.8)	< 0.001
Ever hormone therapy (%)	33,706 (29.4)	2,546 (47.2)	< 0.001
Menopause (%)	73,509 (64.2)	4,939 (91.6)	< 0.001
CHD (%)	2,065 (1.8)	209 (3.9)	< 0.001
Hypertension (%)	21,804 (19.0)	1,371 (25.4)	< 0.001
CKD (%)	787 (0.7)	55 (1.0)	0.005
T1DM (%)	290 (0.3)	31 (0.6)	< 0.001
T2DM (%)	1,367 (1.2)	95 (1.8)	< 0.001
HF (%)	155 (0.1)	12 (0.2)	0.135
Stroke (%)	130 (0.1)	20 (0.4)	< 0.001
Atherosclerosis (%)	41 (0.0)	4 (0.1)	0.287
Parity			< 0.001
0	23,223 (20.3)	952 (17.7)	
1	15,605 (13.6)	689 (12.8)	
2	49,894 (43.5)	2,396 (44.5)	
3	19627 (17.1)	991 (18.4)	
4	4772 (4.2)	265 (4.9)	
5	1458 (1.3)	97 (1.8)	

### Association between parity and osteoporosis

The association between parity and osteoporosis was analyzed using Cox proportional hazards regression (Table 2), with "0 parity" serving as the reference group.

**Table 2:** Association between parity and osteoporosis

	Unweighted		Weighted	
	HR (95% CI)	*P*	HR (95% CI)	*P*
Model 1				
Parity 0	reference		reference	
Parity 1	0.954 (0.864, 1.053)	0.346	0.956 (0.862, 1.060)	0.389
Parity 2	0.906 (0.839, 0.978)	0.012*	0.888 (0.819, 0.963)	0.004**
Parity 3	0.891 (0.814, 0.976)	0.013*	0.888 (0.808, 0.976)	0.014*
Parity 4	0.917 (0.799, 1.052)	0.217	0.911 (0.785, 1.057)	0.218
Parity ≥ 5	1.050 (0.850, 1.297)	0.652	1.252 (1.001, 1.566)	0.049*
Model 2				
Parity 0	reference		reference	
Parity 1	0.947 (0.858, 1.046)	0.283	0.947 (0.854, 1.050)	0.303
Parity 2	0.904 (0.837, 0.976)	0.010*	0.893 (0.823, 0.968)	0.006**
Parity 3	0.885 (0.808, 0.969)	0.008**	0.881 (0.801, 0.968)	0.008**
Parity 4	0.907 (0.790, 1.041)	0.166	0.887 (0.765, 1.029)	0.115
Parity ≥ 5	1.040 (0.842, 1.284)	0.718	0.990 (0.790, 1.240)	0.928
Model 3				
Parity 0	reference		reference	
Parity 1	0.947 (0.858, 1.045)	0.278	0.948 (0.855, 1.052)	0.313
Parity 2	0.904 (0.837, 0.976)	0.010*	0.894 (0.825, 0.970)	0.007**
Parity 3	0.894 (0.817, 0.979)	0.0158*	0.892 (0.811, 0.981)	0.019*
Parity 4	0.919 (0.801, 1.055)	0.232	0.902 (0.777, 1.047)	0.174
Parity ≥ 5	1.063 (0.860, 1.313)	0.572	1.018 (0.812, 1.277)	0.874

Unweighted Model 1 was adjusted for age (continuous), BMI (normal, underweight, overweight, obese), ethnicity (White, Asian, Black, Mixed, Other), education (college or university vs. below college), standard polygenic risk score (PRS) for osteoporosis (continuous), smoking status (never, occasionally, daily or almost daily), alcohol consumption (never/special occasions only, one to three times a month, once or twice a week, three or four times a week, daily or almost daily), diet quality (healthy vs. unhealthy), physical activity level assessed by IPAQ (low, moderate, high), residential area (urban, town, village), employment status (unemployed, student, unpaid or voluntary work, retired, employed or self-employed), and Townsend deprivation index (continuous)

Unweighted Model 2 was additionally adjusted for grip strength (weak vs. not weak), age at menarche (≤ 11, 12–15, ≥ 16 years), and menopausal status (ever vs. never)

Unweighted Model 3 was further adjusted for a history of hysterectomy, bilateral oophorectomy, and hormone therapy, as well as baseline comorbidities, including coronary heart disease (CHD), hypertension, chronic kidney disease (CKD), type 1 diabetes mellitus (T1DM), type 2 diabetes mellitus (T2DM), heart failure (HF), stroke, and atherosclerosis (AS)

Weighted Model 1 was adjusted for parity only

Weighted Model 2 was adjusted for parity and all covariates included in the IPTW estimation model

Weighted Model 3 was additionally adjusted for a history of hysterectomy, bilateral oophorectomy, and baseline comorbidities (CHD, hypertension, CKD, T1DM, T2DM, HF, stroke, and AS)

In the unweighted analysis, both parity 2 and 3 were significantly associated with a reduced risk of osteoporosis across all three models. In the fully adjusted model (Model 3), the hazard ratios (HRs) were 0.904 (95% CI, 0.837–0.976; *P* = 0.010) for parity of 2 and 0.894 (95% CI, 0.817–0.979; *P* = 0.0158) for parity of 3. Parity of 1 and 4 was associated with a protective effect compared to those with no parity, but these differences were not statistically significant. Women with parity ≥ 5 exhibited an HR above 1.00, though this trend did not reach statistical significance.

IPTW-weighted analyses confirmed these protective associations. In the weighted fully adjusted Model 3, parity of 2 yielded an HR of 0.894 (95% CI:0.825–0.970, *P* = 0.007) and parity of 3 showed an HR of 0.892 (95% CI: 0.811–0.981, *P* = 0.019), demonstrating a persistent protective effect against osteoporosis.

We further conducted a sensitivity analysis to explore the association between parity and osteoporosis. This analysis showed that parity of 2 and 3 was associated with a decreased risk of osteoporosis (Supplementary Table 4), similarly to the results of the Cox proportional hazards regression analysis.

### Association between parity and osteoporotic fracture and bone density

A consistent trend toward reduced the risk of osteoporotic fracture was observed with parity < 4. In the unweighted and weighted fully adjusted models, the HRs for parity of 3 were 0.667 (95% CI, 0.472–0.942, *P* = 0.022) and 0.645 (95% CI, 0.449–0.925, *P* = 0.017), respectively (Supplementary Table 5). Sensitivity analysis corroborated this trend, with all parous groups showing HRs below 1.00 compared with nulliparous women. Parity of 3 was associated with a decreased risk of osteoporotic fracture with an HR of 0.673 (95% CI, 0.467–0.969, *P* = 0.033) (Supplementary Table 6). Weighted baseline characteristics between parity groups showed balanced covariate distribution after IPTW (SMD < 0.1; Supplementary Table 7–8; Supplementary Fig. 2).

We next examined the association between parity and bone mineral density; a total of 12,579 participants were included. Because areal BMD reflects bone mineral content rather than bone quality, we examined whether parity was associated with femoral neck or lumbar spine BMD. After adjustment for covariates, the associations were largely non-significant (Table 3). In unweighted Model 1, women who reported two, three, or four live births had lower femoral neck BMD than those who reported none. These associations diminished and were no longer statistically significant after adjustment in Model 2 and Model 3, as well as in all weighted models.

**Table 3:** Association between parity and bone density

	Unweighted		Weighted	
	*β* (95% CI)	*P*	*β* (95% CI)	*P*
**Neck of Femur**				
Model 1				
Parity 0	reference		reference	
Parity 1	−0.008 (−0.017, 0.001)	0.096	−0.004 (−0.013, 0.004)	0.297
Parity 2	−0.013 (−0.020, −0.006)	< 0.001***	0.002 (−0.004, 0.009)	0.493
Parity 3	−0.014 (−0.022, −0.006)	0.001**	0.006 (−0.002, 0.014)	0.157
Parity 4	−0.021 (−0.035, −0.006)	0.005**	−0.002 (−0.016, 0.012)	0.739
Parity ≥ 5	−0.019 (−0.046, 0.008)	0.158	−0.007 (−0.029, 0.015)	0.537
Model 2				
Parity 0	reference		reference	
Parity 1	−0.006 (−0.014, 0.002)	0.157	−0.005 (−0.012, 0.003)	0.231
Parity 2	0.002 (−0.004, 0.008)	0.489	0.003 (−0.003, 0.008)	0.349
Parity 3	0.006 (−0.001, 0.014)	0.106	0.006 (−0.001, 0.013)	0.069
Parity 4	−0.002 (−0.015, 0.011)	0.779	−0.002 (−0.014, 0.010)	0.731
Parity ≥ 5	0.000 (−0.024, 0.024)	0.997	0.000 (−0.020, 0.020)	0.998
Model 3				
Parity 0	reference		reference	
Parity 1	−0.006 (−0.014, 0.002)	0.157	−0.005 (−0.012, 0.003)	0.224
Parity 2	0.002 (−0.004, 0.008)	0.606	0.002 (−0.003, 0.008)	0.436
Parity 3	0.005 (−0.002, 0.013)	0.175	0.005 (−0.001, 0.012)	0.118
Parity 4	−0.002 (−0.015, 0.011)	0.750	−0.003 (−0.014, 0.009)	0.672
Parity ≥ 5	0.001 (−0.023, 0.024)	0.965	0.001 (−0.019, 0.021)	0.942
**Spine**				
Model 1				
Parity 0	reference		reference	
Parity 1	−0.003 (−0.012, 0.006)	0.479	−0.005 (−0.015, 0.005)	0.294
Parity 2	−0.010 (−0.016, −0.003)	0.004**	0.001 (−0.007, 0.008)	0.841
Parity 3	−0.013 (−0.022, −0.005)	0.002**	−0.002 (−0.011, 0.007)	0.633
Parity 4	−0.008 (−0.022, 0.007)	0.296	−0.001 (−0.017, 0.014)	0.863
Parity ≥ 5	−0.002 (−0.029, 0.025)	0.888	0.002 (−0.025, 0.028)	0.902
Model 2				
Parity 0	reference		reference	
Parity 1	−0.006 (−0.013, 0.002)	0.163	−0.005 (−0.014, 0.003)	0.230
Parity 2	0.000 (−0.006, 0.006)	0.973	0.001 (−0.005, 0.008)	0.690
Parity 3	−0.002 (−0.009, 0.006)	0.653	−0.002 (−0.009, 0.006)	0.690
Parity 4	−0.003 (−0.015, 0.010)	0.699	−0.002 (−0.015, 0.011)	0.757
Parity ≥ 5	0.003 (−0.021, 0.026)	0.835	0.001 (−0.023, 0.025)	0.932
Model 3				
Parity 0	reference		reference	
Parity 1	−0.005 (−0.013, 0.002)	0.173	−0.005 (−0.013, 0.003)	0.239
Parity 2	−0.001 (−0.007, 0.005)	0.776	0.000 (−0.006, 0.007)	0.894
Parity 3	−0.003 (−0.010, 0.004)	0.400	−0.003 (−0.011, 0.005)	0.428
Parity 4	−0.003 (−0.016, 0.009)	0.586	−0.003 (−0.016, 0.010)	0.639
Parity ≥ 5	0.003 (−0.020, 0.026)	0.819	0.002 (−0.022, 0.025)	0.900

Unweighted Model 1 was adjusted for age (continuous), BMI (normal, underweight, overweight, obese), ethnicity (White, Asian, Black, Mixed, Other), education (college or university vs. below college), standard polygenic risk score (PRS) for osteoporosis (continuous), smoking status (never, occasionally, daily or almost daily), alcohol consumption (never/special occasions only, one to three times a month, once or twice a week, three or four times a week, daily or almost daily), diet quality (healthy vs. unhealthy), physical activity level assessed by IPAQ (low, moderate, high), residential area (urban, town, village), employment status (unemployed, student, unpaid or voluntary work, retired, employed or self-employed), and Townsend deprivation index (continuous)

Unweighted Model 2 was additionally adjusted for grip strength (weak vs. not weak), age at menarche (≤ 11, 12–15, ≥ 16 years), and menopausal status (ever vs. never).

Unweighted Model 3 was further adjusted for a history of hysterectomy, bilateral oophorectomy, and hormone therapy, as well as baseline comorbidities, including coronary heart disease (CHD), hypertension, chronic kidney disease (CKD), type 1 diabetes mellitus (T1DM), type 2 diabetes mellitus (T2DM), heart failure (HF), stroke, and atherosclerosis (AS).

Weighted Model 1 was adjusted for parity only.

Weighted Model 2 was adjusted for parity and all covariates included in the IPTW estimation model.

Weighted Model 3 was additionally adjusted for a history of hysterectomy, bilateral oophorectomy, and baseline comorbidities (CHD, hypertension, CKD, T1DM, T2DM, HF, stroke, and AS).

### Association between parity and osteoporosis stratified by age and BMI

**Fig. 2:**
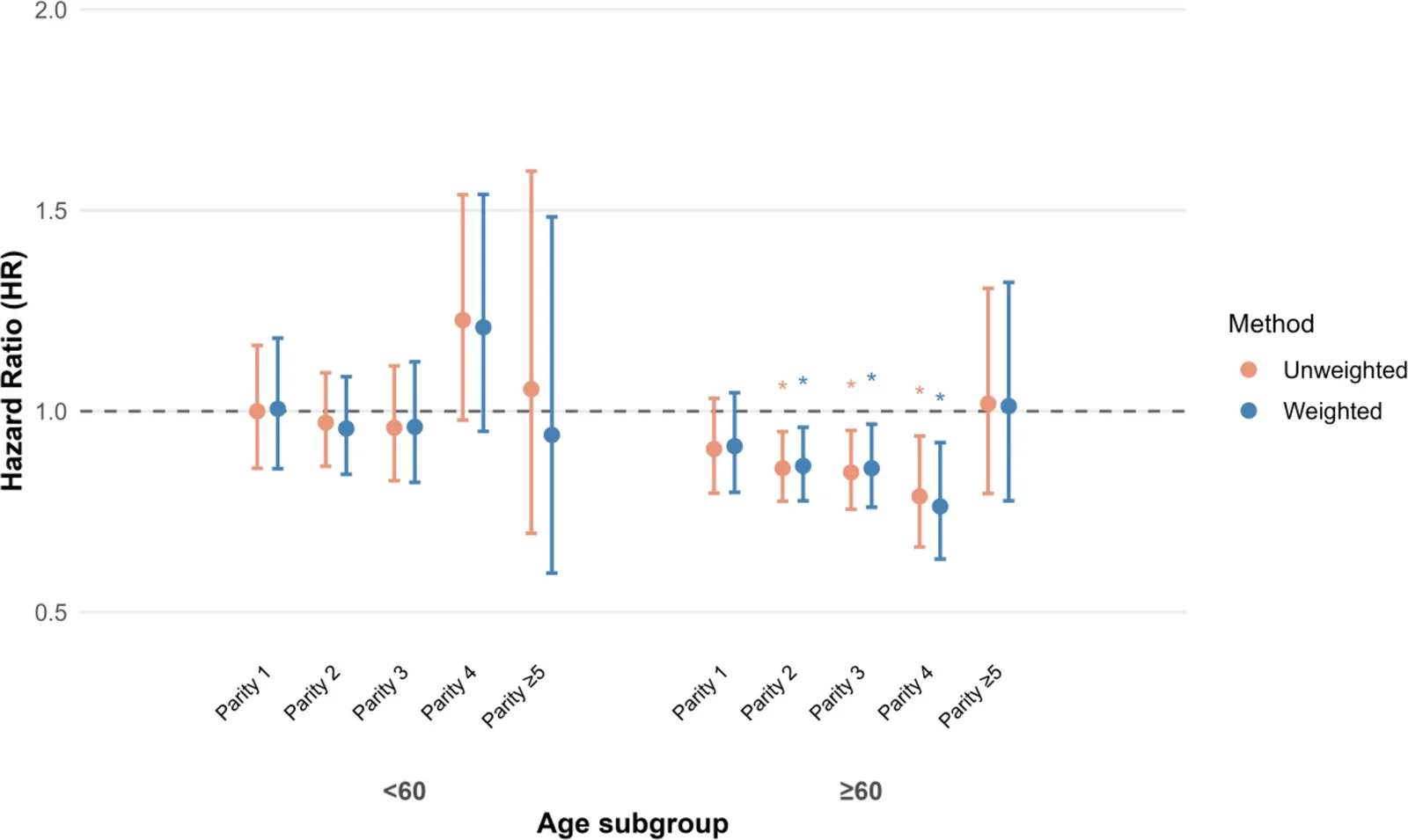
Association between parity and osteoporosis risk stratified by age subgroups. Model adjusted for age, BMI, ethnicity, education, standard osteoporosis PRS, smoking, drinking, diet, IPAQ physical activity level, residential area, employment, Townsend index, grip strength, age of menarche, menopausal status, a history of hysterectomy or bilateral oophorectomy, and baseline comorbidities (CHD, hypertension, CKD, T1DM, T2DM, heart failure, stroke and atherosclerosis). *: *p* < 0.05

Given that age is a key determinant of bone health, we performed a subgroup analysis to assess the association between parity and the risk of osteoporosis across different ages. Employing the World Health Organization (WHO) age threshold of 60 years for defining the elderly, we conducted an analysis to examine the effects of parity on osteoporosis in participants aged < 60 and ≥ 60 years, separately, as shown in Fig. 2. In the < 60 years age group, there was no significant association between the parity and osteoporosis risk. In contrast, a significant inverse association was observed in the older (≥ 60 years) age group. Compared with the parity of 0, parity of 2, 3, and 4 was associated with a statistically significant lower risk of osteoporosis in the ≥ 60 years age group in both unweighted and weighted models.

**Fig. 3:**
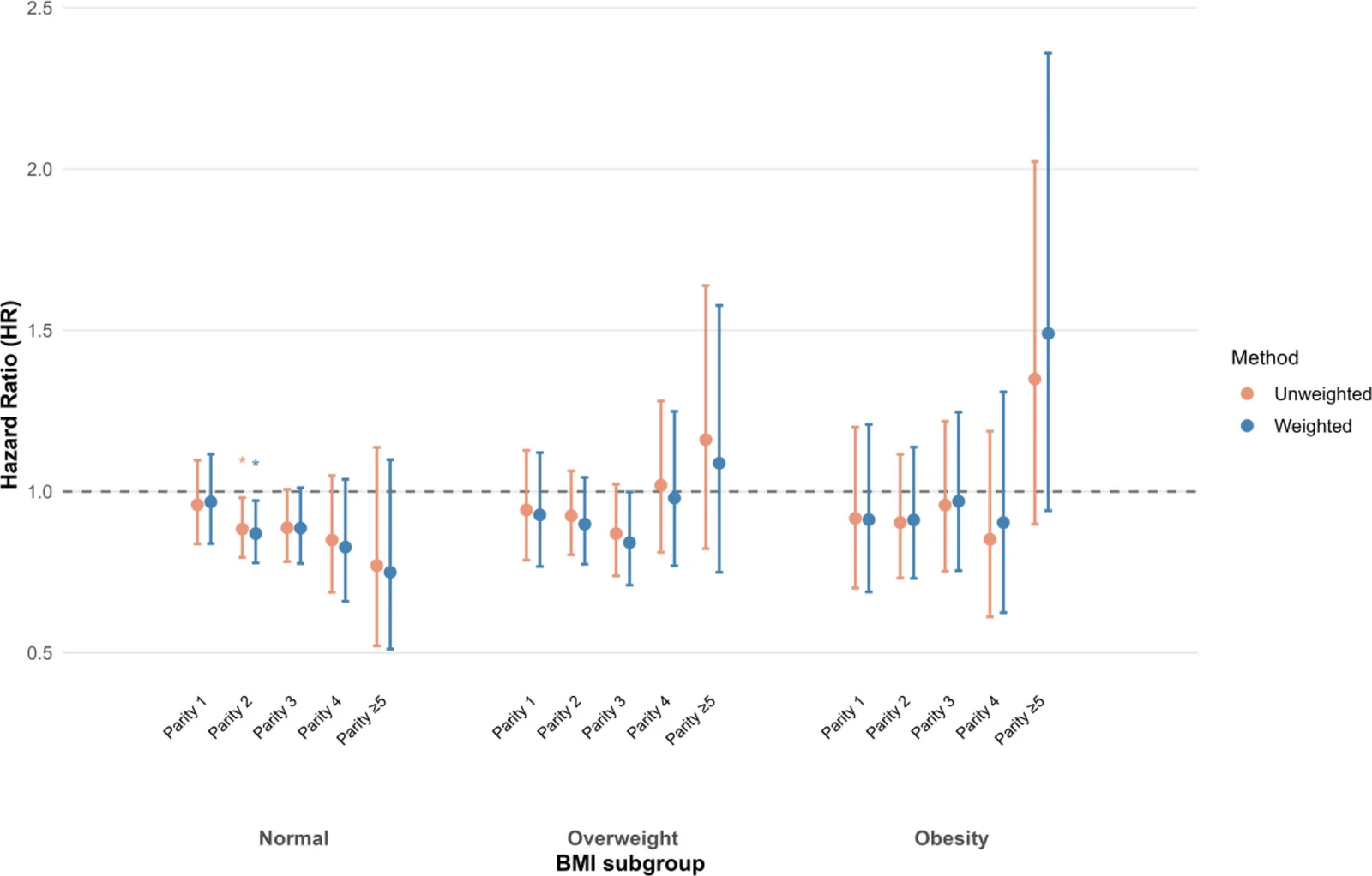
Association between parity and osteoporosis risk in BMI subgroups. Model adjusted for age, BMI, ethnicity, education, Standard osteoporosis PRS, smoking, drinking, diet, IPAQ physical activity level, residential area, employment, Townsend index, grip strength, age of menarche, menopausal status, a history of hysterectomy or bilateral oophorectomy, and baseline comorbidities (CHD, hypertension, CKD, T1DM, T2DM, heart failure, stroke and atherosclerosis). *: *p* < 0.05

In addition, BMI is another key factor in the relationship between parity and osteoporosis. We next performed a subgroup analysis based on BMI. Stratification by BMI indicated that the protective association was largely confined to participants with normal BMI (Fig. 3). In the normal BMI subgroup, parity of 2 was associated with a statistically significant lower risk of osteoporosis in both unweighted and weighted models. Conversely, no significant association was found in the overweight or obesity subgroups.

## Discussion

In this study, we found that parity confers long-term protection against osteoporosis using the UK Biobank cohort. Epidemiological studies have linked multiparity to a reduced risk of hormone-dependent cancers (e.g., ovarian and breast cancer) and autoimmune diseases (e.g., multiple sclerosis) [25–27]. Notably, these diseases are potent risk factors for osteoporosis, and cancer metastasis to the bone can be exacerbated by osteoporosis [28–30]. The concordant risk reduction across multiple organ systems points to a potential bone-initiated crosstalk. Beyond its mechanical role, bone is an active endocrine organ secreting osteokines that can influence other organs and the related diseases [31, 32]. Thus, the parity-induced optimization of the bone may systemically modulate the health of women. While this hypothesis requires direct validation, it positions skeletal health not merely as an outcome, but as a potential contributor to overall physiological resilience.

Although several prior studies have investigated the impact of parity on maternal bone health, their findings remain inconsistent. Regarding bone mineral density (BMD), Seo et al. reported that higher parity was associated with increased low BMD risk in 1,287 Korean women, but their analysis compared multiparous (≥ 3) versus oligoparous (1–2) rather than using a detailed graded approach [11]. Conversely, Egund et al. found no negative BMD impact in 750 women aged 25–35 years [16], yet their sample was smaller and their follow-up was shorter than in our study. Although Gildee and Kramer reported positive associations between parity and BMD in NHANES, their cross-sectional design prevents causal inference and lacks clinical fracture outcomes [17]. When examining hard clinical outcomes like osteoporosis and fracture risk, discrepancies also emerge. Long et al. reported in a meta-analysis that parity ≥ 3 was a predictor of osteoporotic fracture, but heterogeneity across included studies limits comparability [12]. In contrast, Liang et al. found that low parity (0–1) was associated with an increased risk of osteoporosis, though they did not thoroughly examine higher parity (≥ 4) [13]. Wang et al. demonstrated that higher live births were significantly associated with a reduced risk of osteoporotic fractures, though residual statistical heterogeneity could not be fully eliminated [18]. Our study builds upon this evidence through one of the largest single prospective cohorts (n = 119,969). Through a fine-graded parity analysis across categories of 0, 1, 2, 3, 4, and ≥ 5, we revealed a distinct non-linear pattern characterized by statistical protection peaking at 2–3 births and diminishing at ≥ 4. Furthermore, by identifying effect modifications driven by age and BMI, this work offers new insights into the specific subpopulations that benefit most from the skeletal protection of parity.

Our findings suggest that although pregnancy and lactation are periods of physiological calcium loss, higher parity is not associated with an increased risk of postmenopausal osteoporosis; rather, it appears to confer a long-term protective effect at parity of 2 and 3. We proposed that the hormones may optimize bone structure over multiparity [33–35]. The hormonal milieu of pregnancy is characterized by high levels of estrogen, progesterone, and relaxin, which may create a state that strongly inhibits bone resorption and promotes formation [34, 36, 37]. Conversely, lactation presents high levels of parathyroid hormone-related protein (PTHrP), prolactin, and relaxin alongside a low level of estrogen, during which, PTHrP potently stimulates bone formation through sclerostin inhibition, while low estrogen and relaxin levels permit a phase of physiological bone resorption [38, 39]. Based on this dynamic hormonal interplay, we hypothesize that repeated cycles of such remodeling may not only promote microstructural refinements but also induce epigenetic reprogramming in bone marrow mesenchymal and hematopoietic stem cells. These adaptations could lead to alterations in the expression of genes governing the differentiation of osteoblasts and osteoclasts. Thus, we hypothesize that the maternal bone may develop a long-term “memory” of these events, resulting in enhancing bone strength and remodeling capacity.

In addition, repeated cycles of high calcium demand could induce permanent, compensatory adaptations in the maternal calcium regulatory system, including enhanced intestinal absorption and renal reabsorption [40]. We hypothesize that this metabolic advantage may become particularly beneficial in counteracting aging-related calcium imbalance.

In this study, we found that parity was not associated with BMD in the weighted models. This dissociation between the observed protective effect against osteoporosis and the null effect on BMD highlights the limitations of dual-energy X-ray absorptiometry [41]: while BMD measures mineral quantity, it fails to capture important determinants of bone quality, such as microarchitecture and the distinct responses of cortical and trabecular compartments. It has been suggested that parity may compromise trabecular integrity but concurrently promote cortical bone adaptation [19, 21]. Therefore, the findings of this study are not contradictory; rather, they imply that the reduction in osteoporosis risk is unlikely to be mediated by changes in areal BMD and may instead relate to improvements in bone quality or microarchitectural adaptation.

Furthermore, subgroup analyses revealed a significant association between parity of 2 and reduced osteoporosis risk specifically among women with normal BMI. This finding suggests that metabolic status could modulate the bone adaptive response to parity. This protective effect was absent in the overweight and obesity groups, which may be attributable to the regulation of metabolic factors, including chronic inflammatory states and sex hormone metabolism alterations mediated by adipose tissues [42, 43]. These results indicate that metabolic health is essential for realizing the long-term skeletal benefits associated with parity, underscoring the importance of integrating weight management into comprehensive osteoporosis prevention strategies for women.

The strengths of this study include the rigorous design, large sample size and comprehensive adjustment for potential confounders. However, our study still has limitations. First, we lacked measurements of bone microarchitecture and bone turnover markers, which limited our ability to assess the proposed mechanisms. Second, we lacked data on the age at last childbirth in the UK Biobank cohort. This variable is critical because the births occurring closer to the menopausal transition may exert a more profound and long-term influence on maternal bone. Third, the UK Biobank does not contain information on breastfeeding (including its duration and intensity) or infant birth weight. Both factors are known to influence maternal bone turnover and BMD, but their unavailability in our dataset precludes a more detailed analysis of their potential mediating or confounding roles in the observed association. As such, we could not adjust for these variables in the present study. Finally, despite our best efforts to control potential confounders, the unmeasured genetic factors that influence both fertility and bone strength cannot be entirely ruled out.

## Conclusion

In summary, our study demonstrates a protective relationship between parity and long-term bone health. Multiparity (2 and 3) is associated with a reduction in the incidence of osteoporosis in women aged 40–69 years at baseline. This insight opens new avenues for developing strategies to help women build a stronger bone resistant to osteoporosis and osteoporotic fractures across the entire lifespan. Integrating parity into routine fracture-risk algorithms may refine personalized prevention and reduce the lifetime burden of osteoporotic fractures.

## Supplementary Information

Below is the link to the electronic supplementary material.

Supplementary file1 (DOCX 365 KB)

## Data Availability

All UK Biobank information is available online at the webpage www.ukbiobank.ac.uk.
