# Supplementary material for: Non-linear associations between parity and long-term risk of osteoporosis
Source: Arch Osteoporos. 2026 Nov 5;21(1):159. doi: 10.1007/s11657-026-01766-x (PMC13740019; doi:10.1007/s11657-026-01766-x)
Supplement: Supplementary file 1 [file 11657_2026_1766_MOESM1_ESM.docx]

**Non-linear associations between parity and long-term risk of osteoporosis**

Wanqiong Yuan ^1, 2, 3, #^, Jiaojiao Liao ^4, 5, #^, Liyuan Tao ^4, 5, #^, Haoze Song ^6^, Huan Wang ^1, 2, 3^, Jie Huang ^1, 2, 3^, Jinwen He ^1, 2, 3^, Hao Liu ^1, 2, 3^, Tong Wu ^1, 2, 3^, Hao Zhou ^1, 2, 3^, Yundi Zhang ^1, 2, 3^, Chunli Song ^1, 2, 3, *^

^1^ Department of Orthopedics, Peking University Third Hospital, Beijing 100191, China.

^2^ Engineering Research Center of Bone and Joint Precision Medicine, Ministry of Education, Beijing 100191, China

^3^ Beijing Key Laboratory of Advanced Bioadaptable Orthopedic Implants, Beijing 100191, China

^4^ Research Center of Clinical Epidemiology, Peking University Third Hospital, Beijing 100191, China.

^5^ Medical Examination Centre, Peking University Third Hospital, Beijing 100191, China.

^6^ Beijing Normal University，School of Mathematical Science, Beijing 100875, China.

**Supplementary list**

Supplementary table 1. List of field IDs and variable definitions used in the study.

Supplementary table 2. Baseline characteristics of participants grouped by parity.

Supplementary table 3. Weighted baseline characteristics of participants grouped by parity.

Supplementary table 4. Association between parity and osteoporosis by sensitivity analysis.

Supplementary table 5. Association between parity and osteoporotic fracture.

Supplementary table 6. Association between parity and osteoporotic fractures by sensitivity analysis.

Supplementary table 7. Baseline characteristics of participants measured bone density.

Supplementary table 8. Weighted baseline characteristics of participants measured bone density.

Supplementary figure 1. Balance of variables (SMD) across parity before and after IPTW adjustment in the main analysis.

Supplementary figure 2. Balance of variables (SMD) across parity before and after IPTW adjustment in the main analysis in participants with measured bone density.

**Supplementary table 1. List of field IDs and variable definitions used in the study.**

| Field ID | Description | Definition of variable |
| --- | --- | --- |
| 2734 | Number of live births | **Parity:** 1) 0, number of live birth = 0;  2) 1, number of live birth = 1;  3) 2, number of live birth = 2;  4) 3, number of live birth = 3;  5) 4, number of live birth = 4; 6) ≥ 5, number of live birth ≥ 5. |
| 26258 | Standard PRS for osteoporosis (OP) |  |
| 26234 | Standard PRS for estimated bone mineral density t-score (EBMDT) |  |
| 22032 | IPAQ activity group | **IPAQ:** 1) low;  2) moderate;  3) high. |
| 23299 | Femur neck BMD (bone mineral density) (left) | Neck BMD (bone mineral density):  The lower value of left and right neck BMD. |
| 23208 | Femur neck BMD (bone mineral density) (right) |  |
| 23234 | Spine BMD (bone mineral density) |  |
| 21864 | DXA assessment sign-off timestamp |  |
| 46 | Hand grip strength (left) | **Weak grip strength**:  Grip strength of either hand < 18.0 was defined as weak grip strength. |
| 47 | Hand grip strength (right) |  |
| 2714 | Age when periods started (menarche) |  |
| 3591 | Ever had a hysterectomy (womb removed) | **Hysterectomy:** 1) No;  2) Yes. |
| 2834 | Bilateral oophorectomy (both ovaries removed) | **Oophorectomy:** 1) No;  2) Yes. |
| 2814 | Ever used hormone-replacement therapy (HRT) | HRT: 1) No;  2) Yes. |
| 2724 | Had menopause | Menopause: 1) No;  2) Yes. |
| 21000 | Ethnic background |  |
| 21001 | Body mass index (BMI) |  |
| 1239 | Current tobacco smoking | **Smoking:** 1) Never: I have never smoked (1249); 2) Occasionally: Only occasionally (1239), Smoked occasionally (1249), or just tried once or twice (1249); 3) Daily or almost daily: Yes, on most or all days (1239) or smoked on most or all days (1249). |
| 1249 | Past tobacco smoking |  |
| 1558 | Alcohol intake frequency |  |
| 6138 | Qualifications | **Education:** 1) College or University: College or University degree; 2) No: Others. |
| 6142 | Current employment status | **Employment:** Unemployed: Unemployed, looking after home and/or family, or unable to work because of sickness or disability. |
| 20118 | Home area population density-urban or rural | **Area:** 1) Urban: England/Wales - Urban - sparse, England/Wales - Urban - less sparse, Scotland - Large Urban Area, and Scotland - Other Urban Area; 2) Town: England/Wales - Town and Fringe - sparse, England/Wales - Town and Fringe - less sparse, Scotland - Accessible Small Town, Scotland - Remote Small Town, and Scotland - Very Remote Small Town; 3) Village: England/Wales - Village - sparse, England/Wales - Hamlet and Isolated dwelling - sparse, England/Wales - Village - less sparse, England/Wales - Hamlet and Isolated Dwelling - less sparse, Scotland - Accessible Rural, Scotland - Remote Rural, and Scotland - Very Remote Rural. |
| 1289 | Cooked vegetable intake | **Diet:** 1) Healthy: Meet at least 5 of the following patterns: 1. fruits: ≥ 3 servings/day;  2. vegetables: ≥ 3 servings/day;  3. whole grains: ≥ 3 servings/day;  4. refined grains: ≤ 2 servings/day; 5. fish and shellfish: ≥ 2 servings/week;  6. dairy: ≥ 2 servings/day;  7. vegetable oils: ≥ 2 servings/day;  8. processed meats: ≤ 1 serving/week;  9. unprocessed meats: ≤ 2 servings/week;  10. Sugar sweetened beverages (including 100% fruit juice): no consumption; 2) Unhealthy: Others; Notes: Amount of per serving was defined as:  1. fresh fruit: 1 piece;  2. dried fruit: 5 pieces;  3. cooked/raw vegetables: 3 heaped tablespoons; 4. bran/oat/muesli cereal: 1 bowl/day;  5. whole-meal/whole-grain bread: 1 slice/day. |
| 1299 | Salad / raw vegetable intake |  |
| 1309 | Fresh fruit intake |  |
| 1319 | Dried fruit intake |  |
| 1329 | Oily fish intake |  |
| 1339 | Non-oily fish intake |  |
| 1349 | Processed meat intake |  |
| 1359 | Poultry intake |  |
| 1369 | Beef intake |  |
| 1379 | Lamb/mutton intake |  |
| 1389 | Pork intake |  |
| 1408 | Cheese intake |  |
| 1418 | Milk type used |  |
| 1428 | Spread type |  |
| 1438 | Bread intake |  |
| 1448 | Bread type |  |
| 1458 | Cereal intake |  |
| 1468 | Cereal type |  |
| 2654 | Non-butter spread type details |  |
| 6144 | Never eat eggs, dairy, wheat, sugar |  |
| Category 1712 | This category contains data showing the 'first  occurrence' of any code mapped to  3-character ICD-10. | **Ever CVD:** |
|  |  | I20, I21, I22, I23, I24, I25, I60, I61, I62, I63, I64; |
|  |  | **Ever HTN:** |
|  |  | I10, I11, I12, I13, I15; |
|  |  | **Ever CKD:** |
|  |  | N18; |
|  |  | **Ever T1DM:** |
|  |  | E10; |
|  |  | **Ever T2DM:** |
|  |  | E11. |

**Supplementary table 2. Baseline characteristics of participants grouped by parity.**

|  | Parity | | | | | |  |  |
| --- | --- | --- | --- | --- | --- | --- | --- | --- |
|  | 0 | 1 | 2 | 3 | 4 | ≥5 | *P* | *SMD* |
| n | 24175 | 16294 | 52290 | 20618 | 5037 | 1555 |  |  |
| Duration (years, mean (SD)) | 13.36 (1.98) | 13.39 (1.94) | 13.39 (1.96) | 13.36 (2.09) | 13.32 (2.12) | 13.16 (2.36) | <0.001 | 0.01 |
| Age | 52.47 (8.04) | 53.77 (8.20) | 55.61 (7.99) | 56.32 (8.04) | 57.05 (7.98) | 57.46 (8.19) | <0.001 | 0.293 |
| Age >60 (%) | 5779 (23.9) | 4940 (30.3) | 20270 (38.8) | 8626 (41.8) | 2276 (45.2) | 748 (48.1) | <0.001 | 0.238 |
| BMI (mean (SD)) | 26.42 (5.31) | 26.73 (5.19) | 26.41 (4.65) | 26.72 (4.81) | 27.41 (5.22) | 28.48 (5.72) | <0.001 | 0.168 |
| BMI (%) |  |  |  |  |  |  | <0.001 | 0.19 |
| Normal | 11152 (46.1) | 6957 (42.7) | 22758 (43.5) | 8451 (41.0) | 1820 (36.1) | 452 (29.1) |  |  |
| Underweight | 267 (1.1) | 122 (0.7) | 350 (0.7) | 123 (0.6) | 23 (0.5) | 12 (0.8) |  |  |
| Overweight | 7839 (32.4) | 5751 (35.3) | 19413 (37.1) | 7818 (37.9) | 1889 (37.5) | 569 (36.6) |  |  |
| Obesity | 4917 (20.3) | 3464 (21.3) | 9769 (18.7) | 4226 (20.5) | 1305 (25.9) | 522 (33.6) |  |  |
| Ethnicity (%) |  |  |  |  |  |  | <0.001 | 0.196 |
| White | 23003 (95.2) | 15265 (93.7) | 50266 (96.1) | 19547 (94.8) | 4599 (91.3) | 1296 (83.3) |  |  |
| Asian | 391 (1.6) | 339 (2.1) | 843 (1.6) | 397 (1.9) | 145 (2.9) | 59 (3.8) |  |  |
| Black | 334 (1.4) | 337 (2.1) | 547 (1.0) | 364 (1.8) | 186 (3.7) | 143 (9.2) |  |  |
| Mixed | 219 (0.9) | 171 (1.0) | 292 (0.6) | 123 (0.6) | 46 (0.9) | 20 (1.3) |  |  |
| Other | 228 (0.9) | 182 (1.1) | 342 (0.7) | 187 (0.9) | 61 (1.2) | 37 (2.4) |  |  |
| College or University | 11791 (48.8) | 5861 (36.0) | 18140 (34.7) | 7039 (34.1) | 1605 (31.9) | 373 (24.0) | <0.001 | 0.194 |
| Smoking (%) |  |  |  |  |  |  | <0.001 | 0.084 |
| Never | 14906 (61.7) | 9095 (55.8) | 32313 (61.8) | 12425 (60.3) | 2954 (58.6) | 913 (58.7) |  |  |
| Occasionally | 3028 (12.5) | 2212 (13.6) | 7359 (14.1) | 2952 (14.3) | 640 (12.7) | 181 (11.6) |  |  |
| Daily or almost daily | 6241 (25.8) | 4987 (30.6) | 12618 (24.1) | 5241 (25.4) | 1443 (28.6) | 461 (29.6) |  |  |
| Drinking (%) |  |  |  |  |  |  | <0.001 | 0.261 |
| Never | 1786 (7.4) | 1215 (7.5) | 3415 (6.5) | 1719 (8.3) | 635 (12.6) | 323 (20.8) |  |  |
| Special occasions only | 2906 (12.0) | 2306 (14.2) | 6353 (12.1) | 2791 (13.5) | 838 (16.6) | 348 (22.4) |  |  |
| One to three times a month | 3146 (13.0) | 2233 (13.7) | 6500 (12.4) | 2667 (12.9) | 652 (12.9) | 177 (11.4) |  |  |
| Once or twice a week | 5946 (24.6) | 4305 (26.4) | 14466 (27.7) | 5438 (26.4) | 1174 (23.3) | 336 (21.6) |  |  |
| Three or four times a week | 5624 (23.3) | 3471 (21.3) | 12405 (23.7) | 4510 (21.9) | 987 (19.6) | 213 (13.7) |  |  |
| Daily or almost daily | 4767 (19.7) | 2764 (17.0) | 9151 (17.5) | 3493 (16.9) | 751 (14.9) | 158 (10.2) |  |  |
| Healthy diet (%) | 15931 (65.9) | 10070 (61.8) | 33486 (64.0) | 12979 (62.9) | 2980 (59.2) | 891 (57.3) | <0.001 | 0.081 |
| IPAQ (%) |  |  |  |  |  |  | <0.001 | 0.071 |
| Low | 4346 (18.0) | 3096 (19.0) | 8880 (17.0) | 3288 (15.9) | 819 (16.3) | 280 (18.0) |  |  |
| Moderate | 10554 (43.7) | 6938 (42.6) | 22677 (43.4) | 8676 (42.1) | 2048 (40.7) | 602 (38.7) |  |  |
| High | 9275 (38.4) | 6260 (38.4) | 20733 (39.7) | 8654 (42.0) | 2170 (43.1) | 673 (43.3) |  |  |
| Area (%) |  |  |  |  |  |  | <0.001 | 0.093 |
| Urban | 21106 (87.3) | 14214 (87.2) | 44163 (84.5) | 17356 (84.2) | 4356 (86.5) | 1412 (90.8) |  |  |
| Town | 1328 (5.5) | 1011 (6.2) | 4044 (7.7) | 1480 (7.2) | 328 (6.5) | 71 (4.6) |  |  |
| Village | 1741 (7.2) | 1069 (6.6) | 4083 (7.8) | 1782 (8.6) | 353 (7.0) | 72 (4.6) |  |  |
| Employment (%) |  |  |  |  |  |  | <0.001 | 0.267 |
| Unemployed | 1440 (6.0) | 1335 (8.2) | 3847 (7.4) | 1976 (9.6) | 627 (12.4) | 306 (19.7) |  |  |
| Full- or part-time student | 16826 (69.6) | 10703 (65.7) | 31324 (59.9) | 11687 (56.7) | 2653 (52.7) | 685 (44.1) |  |  |
| Doing unpaid or voluntary work | 171 (0.7) | 90 (0.6) | 287 (0.5) | 144 (0.7) | 51 (1.0) | 11 (0.7) |  |  |
| Retired | 122 (0.5) | 81 (0.5) | 156 (0.3) | 90 (0.4) | 25 (0.5) | 11 (0.7) |  |  |
| In paid employment or self-employed | 5616 (23.2) | 4085 (25.1) | 16676 (31.9) | 6721 (32.6) | 1681 (33.4) | 542 (34.9) |  |  |
| Townsend index | -0.80 (3.15) | -1.19 (3.06) | -1.92 (2.70) | -1.61 (2.90) | -0.91 (3.16) | 0.45 (3.60) | <0.001 | 0.307 |
| Menarche age (%) |  |  |  |  |  |  | <0.001 | 0.060 |
| ≤11 | 4681 (19.4) | 3090 (19.0) | 9779 (18.7) | 3739 (18.1) | 907 (18.0) | 282 (18.1) |  |  |
| 12-15 | 18112 (74.9) | 12150 (74.6) | 39684 (75.9) | 15773 (76.5) | 3785 (75.1) | 1142 (73.4) |  |  |
| ≥16 | 1382 (5.7) | 1054 (6.5) | 2827 (5.4) | 1106 (5.4) | 345 (6.8) | 131 (8.4) |  |  |
| Standard PRS (mean (SD)) | -0.06 (0.93) | -0.06 (0.95) | -0.06 (0.92) | -0.06 (0.92) | -0.05 (0.94) | -0.03 (0.95) | 0.816 | 0.013 |
| Weak grip strength (%) | 1487 (6.2) | 1123 (6.9) | 3525 (6.7) | 1574 (7.6) | 420 (8.3) | 142 (9.1) | <0.001 | 0.052 |
| Ever hysterectomy (%) | 837 (3.5) | 820 (5.0) | 3381 (6.5) | 1619 (7.9) | 437 (8.7) | 152 (9.8) | <0.001 | 0.119 |
| Ever bilateral oophorectomy (%) | 570 (2.4) | 405 (2.5) | 1518 (2.9) | 629 (3.1) | 170 (3.4) | 55 (3.5) | <0.001 | 0.034 |
| Ever hormone therapy (%) | 5698 (23.6) | 4633 (28.4) | 16930 (32.4) | 6841 (33.2) | 1676 (33.3) | 474 (30.5) | <0.001 | 0.095 |
| Menopause (%) | 13277 (54.9) | 9839 (60.4) | 35925 (68.7) | 14553 (70.6) | 3700 (73.5) | 1154 (74.2) | <0.001 | 0.195 |
| CHD (%) | 263 (1.1) | 316 (1.9) | 947 (1.8) | 503 (2.4) | 160 (3.2) | 84 (5.4) | <0.001 | 0.104 |
| Hypertension (%) | 4083 (16.9) | 3205 (19.7) | 9983 (19.1) | 4251 (20.6) | 1202 (23.9) | 451 (29.0) | <0.001 | 0.122 |
| CKD (%) | 120 (0.5) | 121 (0.7) | 391 (0.7) | 156 (0.8) | 34 (0.7) | 17 (1.1) | 0.001 | 0.025 |
| T1DM (%) | 81 (0.3) | 63 (0.4) | 114 (0.2) | 42 (0.2) | 19 (0.4) | 2 (0.1) | <0.001 | 0.025 |
| T2DM (%) | 289 (1.2) | 192 (1.2) | 536 (1.0) | 293 (1.4) | 100 (2.0) | 52 (3.3) | <0.001 | 0.068 |
| HF (%) | 21 (0.1) | 38 (0.2) | 61 (0.1) | 31 (0.2) | 11 (0.2) | 5 (0.3) | <0.001 | 0.024 |
| Stroke (%) | 28 (0.1) | 18 (0.1) | 61 (0.1) | 27 (0.1) | 12 (0.2) | 4 (0.3) | 0.149 | 0.018 |
| Atherosclerosis (%) | 7 (0.0) | 5 (0.0) | 20 (0.0) | 10 (0.0) | 3 (0.1) | 0 (0.0) | 0.764 | 0.015 |

**Supplementary table 3. Weighted baseline characteristics of participants grouped by parity.**

|  | Parity |  |  |  |  |  |  |  |
| --- | --- | --- | --- | --- | --- | --- | --- | --- |
|  | 0 | 1 | 2 | 3 | 4 | ≥5 |  | *SMD* |
| n | 120748.57 | 120038.13 | 119906.03 | 119823.64 | 116529.9 | 52427.33 |  |  |
| Age | 54.95 (8.05) | 54.96 (8.21) | 54.94 (8.14) | 54.95 (8.28) | 55.19 (8.17) | 57.23 (8.19) | <0.001 | 0.099 |
| BMI (mean (SD)) | 26.60 (5.18) | 26.64 (5.07) | 26.55 (4.82) | 26.57 (4.86) | 26.58 (4.91) | 27.54 (5.28) | <0.001 | 0.068 |
| BMI (%) |  |  |  |  |  |  | <0.001 | 0.073 |
| Normal | 52200.1 (43.2) | 51365.1 (42.8) | 51447.1 (42.9) | 51738.7 (43.2) | 50481.7 (43.3) | 18083.0 (34.5) |  |  |
| Underweight | 913.6 (0.8) | 913.6 (0.8) | 921.4 (0.8) | 906.7 (0.8) | 738.1 (0.6) | 423.1 (0.8) |  |  |
| Overweight | 43109.1 (35.7) | 43434.1 (36.2) | 43294.0 (36.1) | 43089.0 (36.0) | 42055.3 (36.1) | 20014.5 (38.2) |  |  |
| Obesity | 24525.7 (20.3) | 24325.3 (20.3) | 24243.6 (20.2) | 24089.2 (20.1) | 23254.9 (20.0) | 13906.7 (26.5) |  |  |
| Ethnicity (%) |  |  |  |  |  |  | 0.001 | 0.037 |
| White | 114607.8 (94.9) | 114041.9 (95.0) | 113850.3 (94.9) | 113792.5 (95.0) | 110825.6 (95.1) | 48748.4 (93.0) |  |  |
| Asian | 2200.2 (1.8) | 2215.6 (1.8) | 2160.7 (1.8) | 2185.2 (1.8) | 2040.8 (1.8) | 1144.8 (2.2) |  |  |
| Black | 1955.4 (1.6) | 1870.7 (1.6) | 1958.8 (1.6) | 1949.2 (1.6) | 1871.4 (1.6) | 1490.4 (2.8) |  |  |
| Mixed | 880.4 (0.7) | 884.0 (0.7) | 879.2 (0.7) | 864.8 (0.7) | 765.7 (0.7) | 504.4 (1.0) |  |  |
| Other | 1104.8 (0.9) | 1025.9 (0.9) | 1057.0 (0.9) | 1031.9 (0.9) | 1026.4 (0.9) | 539.3 (1.0) |  |  |
| College or University | 44376.0 (36.8) | 44513.7 (37.1) | 44667.2 (37.3) | 44702.8 (37.3) | 43766.5 (37.6) | 14711.5 (28.1) | <0.001 | 0.070 |
| Smoking (%) |  |  |  |  |  |  | 0.007 | 0.036 |
| Never | 72762.7 (60.3) | 72659.3 (60.5) | 72462.5 (60.4) | 72549.0 (60.5) | 69713.5 (59.8) | 29857.4 (57.0) |  |  |
| Occasionally | 16447.2 (13.6) | 16317.8 (13.6) | 16352.2 (13.6) | 16292.0 (13.6) | 15953.3 (13.7) | 6705.1 (12.8) |  |  |
| Daily or almost daily | 31538.7 (26.1) | 31061.0 (25.9) | 31091.3 (25.9) | 30982.6 (25.9) | 30863.1 (26.5) | 15864.8 (30.3) |  |  |
| Drinking (%) |  |  |  |  |  |  | <0.001 | 0.107 |
| Never | 9177.4 (7.6) | 9181.5 (7.6) | 9115.8 (7.6) | 9176.1 (7.7) | 8938.2 (7.7) | 7032.4 (13.4) |  |  |
| Special occasions only | 16151.2 (13.4) | 15517.0 (12.9) | 15529.6 (13.0) | 15596.1 (13.0) | 15336.2 (13.2) | 10043.9 (19.2) |  |  |
| One to three times a month | 15416.5 (12.8) | 15335.2 (12.8) | 15362.4 (12.8) | 15303.6 (12.8) | 14779.9 (12.7) | 6901.6 (13.2) |  |  |
| Once or twice a week | 31665.9 (26.2) | 31640.9 (26.4) | 31630.3 (26.4) | 31598.3 (26.4) | 30461.4 (26.1) | 13054.8 (24.9) |  |  |
| Three or four times a week | 27086.6 (22.4) | 27379.1 (22.8) | 27172.4 (22.7) | 27145.1 (22.7) | 26587.2 (22.8) | 8758.1 (16.7) |  |  |
| Daily or almost daily | 21250.9 (17.6) | 20984.5 (17.5) | 21095.6 (17.6) | 21004.5 (17.5) | 20426.9 (17.5) | 6636.5 (12.7) |  |  |
| Healthy diet (%) | 76237.0 (63.1) | 76250.7 (63.5) | 76195.3 (63.5) | 76118.8 (63.5) | 73536.1 (63.1) | 31531.2 (60.1) | 0.031 | 0.026 |
| IPAQ (%) |  |  |  |  |  |  | 0.025 | 0.033 |
| Low | 21042.0 (17.4) | 20818.6 (17.3) | 20702.5 (17.3) | 20707.4 (17.3) | 19889.4 (17.1) | 9150.7 (17.5) |  |  |
| Moderate | 51787.5 (42.9) | 51193.7 (42.6) | 51480.7 (42.9) | 51113.9 (42.7) | 49797.6 (42.7) | 20317.9 (38.8) |  |  |
| High | 47919.1 (39.7) | 48025.8 (40.0) | 47722.8 (39.8) | 48002.4 (40.1) | 46843.0 (40.2) | 22958.7 (43.8) |  |  |
| Area (%) |  |  |  |  |  |  | 0.013 | 0.038 |
| Urban | 102952.9 (85.3) | 102691.7 (85.5) | 102492.5 (85.5) | 102429.3 (85.5) | 99443.5 (85.3) | 46504.6 (88.7) |  |  |
| Town | 8491.5 (7.0) | 8217.1 (6.8) | 8275.3 (6.9) | 8230.5 (6.9) | 7898.5 (6.8) | 2924.2 (5.6) |  |  |
| Village | 9304.2 (7.7) | 9129.3 (7.6) | 9138.2 (7.6) | 9163.9 (7.6) | 9188.0 (7.9) | 2998.6 (5.7) |  |  |
| Employment (%) |  |  |  |  |  |  | <0.001 | 0.098 |
| Unemployed | 10599.6 (8.8) | 9522.1 (7.9) | 9527.4 (7.9) | 9823.6 (8.2) | 10068.4 (8.6) | 7574.2 (14.4) |  |  |
| Full or part-time student | 73484.3 (60.9) | 73857.4 (61.5) | 73838.4 (61.6) | 73590.0 (61.4) | 71443.9 (61.3) | 26263.1 (50.1) |  |  |
| Doing unpaid or voluntary work | 797.2 (0.7) | 748.8 (0.6) | 750.0 (0.6) | 771.8 (0.6) | 703.5 (0.6) | 416.4 (0.8) |  |  |
| Retired | 522.5 (0.4) | 485.2 (0.4) | 485.0 (0.4) | 496.1 (0.4) | 486.5 (0.4) | 342.5 (0.7) |  |  |
| In paid employment or self-employed | 35345.0 (29.3) | 35424.8 (29.5) | 35305.3 (29.4) | 35142.2 (29.3) | 33827.6 (29.0) | 17831.1 (34.0) |  |  |
| Townsend index | -1.51 (2.92) | -1.46 (2.97) | -1.47 (2.97) | -1.48 (2.98) | -1.47 (2.91) | -0.50 (3.23) | <0.001 | 0.111 |
| Menarche age (%) |  |  |  |  |  |  | 0.876 | 0.015 |
| ≤11 | 22336.5 (18.5) | 22629.3 (18.9) | 22370.0 (18.7) | 22356.1 (18.7) | 21254.3 (18.2) | 10053.7 (19.2) |  |  |
| 12-15 | 91448.1 (75.7) | 90486.7 (75.4) | 90689.3 (75.6) | 90592.4 (75.6) | 88562.5 (76.0) | 39070.3 (74.5) |  |  |
| ≥16 | 6964.0 (5.8) | 6922.1 (5.8) | 6846.8 (5.7) | 6875.1 (5.7) | 6713.1 (5.8) | 3303.3 (6.3) |  |  |
| Standard PRS (mean (SD)) | -0.06 (0.93) | -0.06 (0.95) | -0.06 (0.92) | -0.06 (0.92) | -0.06 (0.94) | -0.04 (0.94) | 0.968 | 0.008 |
| Weak grip strength (%) | 8382.9 (6.9) | 8329.4 (6.9) | 8257.1 (6.9) | 8239.9 (6.9) | 7868.5 (6.8) | 4409.6 (8.4) | 0.058 | 0.022 |

**Supplementary table 4. Association between parity and osteoporosis by sensitivity analysis**

|  | Unweighted |  |  | Weighted |  |
| --- | --- | --- | --- | --- | --- |
|  | HR (95% CI) | *P* |  | HR (95% CI) | *P* |
| Model 1 |  |  |  |  |  |
| Parity 0 | reference |  |  | reference |  |
| Parity 1 | 0.974 (0.879, 1.078) | 0.610 |  | 0.968 (0.868, 1.080) | 0.565 |
| Parity 2 | 0.913 (0.843, 0.989) | 0.026* |  | 0.905 (0.830, 0.987) | 0.023* |
| Parity 3 | 0.886 (0.806, 0.973) | 0.012* |  | 0.890 (0.805, 0.984) | 0.023* |
| Parity 4 | 0.912 (0.790, 1.052) | 0.206 |  | 0.904 (0.773, 1.056) | 0.204 |
| Parity ≥5 | 1.030 (0.826, 1.284) | 0.794 |  | 0.982 (0.775, 1.245) | 0.881 |
| Model 2 |  |  |  |  |  |
| Parity 0 | reference |  |  | reference |  |
| Parity 1 | 0.977 (0.882, 1.082) | 0.651 |  | 0.967 (0.867, 1.078) | 0.542 |
| Parity 2 | 0.917 (0.847, 0.994) | 0.034* |  | 0.900 (0.826, 0.982) | 0.017* |
| Parity 3 | 0.898 (0.817, 0.987) | 0.026* |  | 0.889 (0.804, 0.983) | 0.022* |
| Parity 4 | 0.929 (0.805, 1.072) | 0.312 |  | 0.903 (0.773, 1.056) | 0.201 |
| Parity ≥5 | 1.060 (0.850, 1.321) | 0.606 |  | 0.996 (0.785, 1.264) | 0.976 |
| Model 3 |  |  |  |  |  |
| Parity 0 | reference |  |  | reference |  |
| Parity 1 | 0.971 (0.876, 1.075) | 0.566 |  | 0.964 (0.865, 1.076) | 0.515 |
| Parity 2 | 0.915 (0.845, 0.992) | 0.030* |  | 0.899 (0.825, 0.980) | 0.016* |
| Parity 3 | 0.896 (0.815, 0.985) | 0.023* |  | 0.888 (0.803, 0.981) | 0.020* |
| Parity 4 | 0.924 (0.800, 1.066) | 0.277 |  | 0.903 (0.773, 1.056) | 0.202 |
| Parity ≥5 | 1.056 (0.847, 1.317) | 0.628 |  | 1.000 (0.789, 1.268) | 0.999 |

**Supplementary table 4.** Unweighted Model 1 Adjusted by covariates age (continuous), BMI (normal, underweight, overweight, obese), ethnicity (White, Asian, Black, Mixed, Other), education (college or university, not), Standard osteoporosis PRS (continuous), smoking (never, occasionally, daily or almost daily), drinking (Never special occasions only, One to three times a month, Once or twice a week, Three or four times a week, Daily or almost daily), diet (healthy, unhealthy), IPAQ (low, moderate, high), area (urban, town, village), employment (Unemployed, Full or part-time student, Doing unpaid or voluntary work, Retired, In paid employment or self-employed), Townsend index (continuous).

Unweighted Model 2 was additionally adjusted for grip strength (weak vs. not weak), age at menarche (≤11, 12-15, ≥16 years), and menopausal status (ever vs. never).

Unweighted Model 3 was further adjusted for a history of hysterectomy, bilateral oophorectomy, and hormone therapy, as well as baseline comorbidities, including coronary heart disease (CHD), hypertension, chronic kidney disease (CKD), type 1 diabetes mellitus (T1DM), type 2 diabetes mellitus (T2DM), heart failure (HF), stroke, and atherosclerosis (AS).

Weighted Model 1 was adjusted for parity only.
Weighted Model 2 was adjusted for parity and all covariates included in the IPTW estimation model.
Weighted Model 3 was additionally adjusted for a history of hysterectomy, bilateral oophorectomy, and baseline comorbidities (CHD, hypertension, CKD, T1DM, T2DM, HF, stroke, and AS).

**Supplementary table 5. Association between parity and osteoporotic fracture**

|  | Unweighted |  |  | Weighted |  |
| --- | --- | --- | --- | --- | --- |
|  | HR (95% CI) | *P* |  | HR (95% CI) | *P* |
| Model 1 |  |  |  |  |  |
| Parity 0 | reference |  |  | reference |  |
| Parity 1 | 0.976 (0.675, 1.411) | 0.898 |  | 0.804 (0.547, 1.181) | 0.265 |
| Parity 2 | 1.240 (0.945, 1.628) | 0.121 |  | 0.875 (0.655, 1.170) | 0.368 |
| Parity 3 | 1.016 (0.723, 1.428) | 0.928 |  | 0.662 (0.464, 0.945) | 0.023* |
| Parity 4 | 1.752 (1.118, 2.746) | 0.014* |  | 0.964 (0.598, 1.552) | 0.879 |
| Parity ≥5 | 2.211 (1.141, 4.287) | 0.019 |  | 1.595 (0.790, 3.217) | 0.193 |
| Model 2 |  |  |  |  |  |
| Parity 0 | reference |  |  | reference |  |
| Parity 1 | 0.798 (0.551, 1.156) | 0.232 |  | 0.779 (0.531, 1.142) | 0.201 |
| Parity 2 | 0.910 (0.689, 1.200) | 0.503 |  | 0.870 (0.652, 1.161) | 0.345 |
| Parity 3 | 0.663 (0.469, 0.936) | 0.019* |  | 0.637 (0.446, 0.910) | 0.013* |
| Parity 4 | 0.996 (0.632, 1.569) | 0.986 |  | 0.890 (0.553, 1.434) | 0.633 |
| Parity ≥5 | 1.119 (0.573, 2.185) | 0.743 |  | 1.089 (0.541, 2.190) | 0.811 |
| Model 3 |  |  |  |  |  |
| Parity 0 | reference |  |  | reference |  |
| Parity 1 | 0.794 (0.548, 1.150) | 0.222 |  | 0.775 (0.527, 1.138) | 0.193 |
| Parity 2 | 0.908 (0.688, 1.199) | 0.497 |  | 0.870 (0.650, 1.165) | 0.351 |
| Parity 3 | 0.667 (0.472, 0.942) | 0.022* |  | 0.645 (0.449, 0.925) | 0.017* |
| Parity 4 | 1.005 (0.637, 1.584) | 0.984 |  | 0.906 (0.564, 1.455) | 0.683 |
| Parity ≥5 | 1.121 (0.573, 2.191) | 0.739 |  | 1.083 (0.529, 2.215) | 0.828 |

**Supplementary table 5.** Unweighted Model 1 was adjusted for age (continuous), BMI (normal, underweight, overweight, obese), ethnicity (White, Asian, Black, Mixed, Other), education (college or university vs. below college), standard polygenic risk score (PRS) for osteoporosis (continuous), smoking status (never, occasionally, daily or almost daily), alcohol consumption (never/special occasions only, one to three times a month, once or twice a week, three or four times a week, daily or almost daily), diet quality (healthy, unhealthy), physical activity level assessed by IPAQ (low, moderate, high), residential area (urban, town, village), employment status (unemployed, student, unpaid or voluntary work, retired, employed or self-employed), and Townsend deprivation index (continuous).

Unweighted Model 2 was additionally adjusted for grip strength (weak vs. not weak), age at menarche (≤11, 12–15, ≥16 years), and menopausal status (ever vs. never).

Unweighted Model 3 was further adjusted for a history of hysterectomy, bilateral oophorectomy, and hormone therapy, as well as baseline comorbidities, including coronary heart disease (CHD), hypertension, chronic kidney disease (CKD), type 1 diabetes mellitus (T1DM), type 2 diabetes mellitus (T2DM), heart failure (HF), stroke, and atherosclerosis (AS).

Weighted Model 1 was adjusted for parity only.
Weighted Model 2 was adjusted for parity and all covariates included in the IPTW estimation model.
Weighted Model 3 was additionally adjusted for a history of hysterectomy, bilateral oophorectomy, and baseline comorbidities (CHD, hypertension, CKD, T1DM, T2DM, HF, stroke, and AS).

**Supplementary table 6. Association between parity and osteoporotic fracture by sensitivity analysis**

|  | Unweighted |  |  | Weighted |  |
| --- | --- | --- | --- | --- | --- |
|  | HR (95% CI) | *P* |  | HR (95% CI) | *P* |
| Model 1 |  |  |  |  |  |
| Parity 0 | reference |  |  | reference |  |
| Parity 1 | 0.792 (0.543, 1.154) | 0.225 |  | 0.776 (0.523, 1.152) | 0.209 |
| Parity 2 | 0.904 (0.682, 1.198) | 0.482 |  | 0.890 (0.661, 1.199) | 0.444 |
| Parity 3 | 0.676 (0.477, 0.959) | 0.028* |  | 0.675 (0.469, 0.973) | 0.035* |
| Parity 4 | 1.040 (0.659, 1.641) | 0.867 |  | 0.958 (0.593, 1.548) | 0.861 |
| Parity ≥5 | 1.048 (0.520, 2.115) | 0.895 |  | 0.990 (0.471, 2.080) | 0.979 |
| Model 2 |  |  |  |  |  |
| Parity 0 | reference |  |  | reference |  |
| Parity 1 | 0.789 (0.541, 1.150) | 0.217 |  | 0.774 (0.522, 1.148) | 0.202 |
| Parity 2 | 0.904 (0.682, 1.198) | 0.482 |  | 0.882 (0.655, 1.188) | 0.408 |
| Parity 3 | 0.679 (0.479, 0.963) | 0.030* |  | 0.671 (0.466, 0.967) | 0.032* |
| Parity 4 | 1.043 (0.661, 1.646) | 0.856 |  | 0.955 (0.592, 1.542) | 0.851 |
| Parity ≥5 | 1.055 (0.523, 2.129) | 0.882 |  | 0.993 (0.472, 2.088) | 0.984 |
| Model 3 |  |  |  |  |  |
| Parity 0 | reference |  |  | reference |  |
| Parity 1 | 0.786 (0.539, 1.146) | 0.211 |  | 0.776 (0.523, 1.151) | 0.207 |
| Parity 2 | 0.907 (0.684, 1.202) | 0.496 |  | 0.882 (0.655, 1.188) | 0.409 |
| Parity 3 | 0.683 (0.482, 0.969) | 0.032* |  | 0.673 (0.467, 0.969) | 0.033* |
| Parity 4 | 1.046 (0.662, 1.652) | 0.848 |  | 0.956 (0.593, 1.541) | 0.853 |
| Parity ≥5 | 1.045 (0.517, 2.112) | 0.901 |  | 0.985 (0.466, 2.083) | 0.968 |

**Supplementary table 6.** Unweighted Model 1 Adjusted by covariates age (continuous), BMI (normal, underweight, overweight, obese), ethnicity (White, Asian, Black, Mixed, Other), education (college or university, not), Standard osteoporosis PRS (continuous), smoking (never, occasionally, daily or almost daily), drinking (Never Special occasions only, One to three times a month, Once or twice a week, Three or four times a week, Daily or almost daily), diet (healthy, unhealthy), IPAQ (low, moderate, high), area (urban, town, village), employment (Unemployed, Full or part-time student, Doing unpaid or voluntary work, Retired, In paid employment or self-employed), Townsend index (continuous).

Unweighted Model 2 was additionally adjusted for grip strength (weak vs. not weak), age at menarche (≤11, 12-15, ≥16 years), and menopausal status (ever vs. never).

Unweighted Model 3 was further adjusted for a history of hysterectomy, bilateral oophorectomy, and hormone therapy, as well as baseline comorbidities, including coronary heart disease (CHD), hypertension, chronic kidney disease (CKD), type 1 diabetes mellitus (T1DM), type 2 diabetes mellitus (T2DM), heart failure (HF), stroke, and atherosclerosis (AS).

Weighted Model 1 was adjusted for parity only.
Weighted Model 2 was adjusted for parity and all covariates included in the IPTW estimation model.
Weighted Model 3 was additionally adjusted for was additionally adjusted for a history of hysterectomy, bilateral oophorectomy, and baseline comorbidities (CHD, hypertension, CKD, T1DM, T2DM, HF, stroke, and AS).

**Supplementary table 7. Baseline characteristics of participants measured bone density.**

|  | Parity | | | | | | *P* | *SMD* |
| --- | --- | --- | --- | --- | --- | --- | --- | --- |
|  | 0 | 1 | 2 | 3 | 4 | ≥5 |  |  |
| n | 2825 | 1596 | 5481 | 2089 | 467 | 121 |  |  |
| BMD (Neck of Femur) | 0.89 (0.13) | 0.88 (0.13) | 0.87 (0.13) | 0.87 (0.13) | 0.87 (0.13) | 0.87 (0.12) | 0.001 | 0.063 |
| BMD (Spine) | 1.01 (0.15) | 1.01 (0.15) | 1.00 (0.15) | 1.00 (0.15) | 1.00 (0.15) | 1.01 (0.15) | 0.019 | 0.043 |
| Age | 60.76 (7.48) | 61.59 (7.49) | 63.25 (7.75) | 64.14 (7.68) | 65.11 (7.84) | 64.67 (7.63) | <0.001 | 0.278 |
| BMI (mean (SD)) | 25.72 (4.75) | 26.08 (4.61) | 25.67 (4.13) | 25.81 (4.29) | 26.36 (4.51) | 26.94 (4.63) | <0.001 | 0.126 |
| BMI (%) |  |  |  |  |  |  | <0.001 | 0.155 |
| Normal | 1462 (51.8) | 757 (47.4) | 2744 (50.1) | 1020 (48.8) | 205 (43.9) | 46 (38.0) |  |  |
| Underweight | 31 (1.1) | 12 (0.8) | 34 (0.6) | 16 (0.8) | 2 (0.4) | 2 (1.7) |  |  |
| Overweight | 894 (31.6) | 562 (35.2) | 1945 (35.5) | 748 (35.8) | 169 (36.2) | 45 (37.2) |  |  |
| Obesity | 438 (15.5) | 265 (16.6) | 758 (13.8) | 305 (14.6) | 91 (19.5) | 28 (23.1) |  |  |
| Ethnicity (%) |  |  |  |  |  |  | 0.036 | 0.105 |
| White | 2725 (96.5) | 1529 (95.8) | 5323 (97.1) | 2011 (96.3) | 451 (96.6) | 116 (95.9) |  |  |
| Asian | 34 (1.2) | 25 (1.6) | 72 (1.3) | 30 (1.4) | 2 (0.4) | 1 (0.8) |  |  |
| Black | 22 (0.8) | 12 (0.8) | 37 (0.7) | 19 (0.9) | 9 (1.9) | 1 (0.8) |  |  |
| Mixed | 25 (0.9) | 15 (0.9) | 25 (0.5) | 10 (0.5) | 3 (0.6) | 1 (0.8) |  |  |
| Other | 19 (0.7) | 15 (0.9) | 24 (0.4) | 19 (0.9) | 2 (0.4) | 2 (1.7) |  |  |
| College or University | 1637 (57.9) | 717 (44.9) | 2548 (46.5) | 981 (47.0) | 199 (42.6) | 51 (42.1) | <0.001 | 0.126 |
| Smoking (%) |  |  |  |  |  |  | <0.001 | 0.102 |
| Never | 1861 (65.9) | 943 (59.1) | 3532 (64.4) | 1303 (62.4) | 295 (63.2) | 75 (62.0) |  |  |
| Occasionally | 351 (12.4) | 215 (13.5) | 767 (14.0) | 304 (14.6) | 62 (13.3) | 22 (18.2) |  |  |
| Daily or almost daily | 613 (21.7) | 438 (27.4) | 1182 (21.6) | 482 (23.1) | 110 (23.6) | 24 (19.8) |  |  |
| Drinking (%) |  |  |  |  |  |  | <0.001 | 0.247 |
| Never | 162 (5.7) | 69 (4.3) | 228 (4.2) | 119 (5.7) | 34 (7.3) | 17 (14.0) |  |  |
| Special occasions only | 262 (9.3) | 194 (12.2) | 528 (9.6) | 205 (9.8) | 52 (11.1) | 25 (20.7) |  |  |
| One to three times a month | 365 (12.9) | 229 (14.3) | 674 (12.3) | 248 (11.9) | 60 (12.8) | 19 (15.7) |  |  |
| Once or twice a week | 710 (25.1) | 425 (26.6) | 1502 (27.4) | 556 (26.6) | 110 (23.6) | 21 (17.4) |  |  |
| Three or four times a week | 745 (26.4) | 385 (24.1) | 1550 (28.3) | 551 (26.4) | 120 (25.7) | 26 (21.5) |  |  |
| Daily or almost daily | 581 (20.6) | 294 (18.4) | 999 (18.2) | 410 (19.6) | 91 (19.5) | 13 (10.7) |  |  |
| Healthy diet (%) | 1955 (69.2) | 1018 (63.8) | 3552 (64.8) | 1363 (65.2) | 292 (62.5) | 78 (64.5) | <0.001 | 0.054 |
| IPAQ (%) |  |  |  |  |  |  | <0.001 | 0.147 |
| Low | 499 (17.7) | 325 (20.4) | 932 (17.0) | 348 (16.7) | 66 (14.1) | 24 (19.8) |  |  |
| Moderate | 1283 (45.4) | 706 (44.2) | 2422 (44.2) | 858 (41.1) | 181 (38.8) | 42 (34.7) |  |  |
| High | 1043 (36.9) | 565 (35.4) | 2127 (38.8) | 883 (42.3) | 220 (47.1) | 55 (45.5) |  |  |
| Area (%) |  |  |  |  |  |  | 0.302 | 0.045 |
| Urban | 2429 (86.0) | 1348 (84.5) | 4585 (83.7) | 1754 (84.0) | 400 (85.7) | 102 (84.3) |  |  |
| Town | 174 (6.2) | 113 (7.1) | 433 (7.9) | 149 (7.1) | 33 (7.1) | 8 (6.6) |  |  |
| Village | 222 (7.9) | 135 (8.5) | 463 (8.4) | 186 (8.9) | 34 (7.3) | 11 (9.1) |  |  |
| Employment (%) |  |  |  |  |  |  | <0.001 | 0.221 |
| Unemployed | 120 (4.2) | 90 (5.6) | 378 (6.9) | 178 (8.5) | 47 (10.1) | 17 (14.0) |  |  |
| Full- or part-time student | 2148 (76.0) | 1189 (74.5) | 3760 (68.6) | 1410 (67.5) | 301 (64.5) | 80 (66.1) |  |  |
| Doing unpaid or voluntary work | 25 (0.9) | 11 (0.7) | 34 (0.6) | 14 (0.7) | 3 (0.6) | 4 (3.3) |  |  |
| Retired | 14 (0.5) | 7 (0.4) | 22 (0.4) | 11 (0.5) | 1 (0.2) | 0 (0.0) |  |  |
| In paid employment or self-employed | 518 (18.3) | 299 (18.7) | 1287 (23.5) | 476 (22.8) | 115 (24.6) | 20 (16.5) |  |  |
| Townsend index | -1.11 (3.05) | -1.49 (2.86) | -2.21 (2.50) | -1.95 (2.58) | -1.63 (2.79) | -0.90 (3.26) | <0.001 | 0.215 |
| Menarche age (%) |  |  |  |  |  |  | 0.564 | 0.057 |
| ≤11 | 521 (18.4) | 286 (17.9) | 958 (17.5) | 359 (17.2) | 72 (15.4) | 22 (18.2) |  |  |
| 12-15 | 2161 (76.5) | 1210 (75.8) | 4242 (77.4) | 1630 (78.0) | 369 (79.0) | 91 (75.2) |  |  |
| ≥16 | 143 (5.1) | 100 (6.3) | 281 (5.1) | 100 (4.8) | 26 (5.6) | 8 (6.6) |  |  |
| S_PRS_EBMDT (mean (SD)) | -0.04 (0.97) | 0.00 (0.98) | -0.01 (0.97) | 0.02 (0.95) | 0.07 (0.99) | 0.04 (0.96) | 0.105 | 0.05 |
| Weak grip strength (%) | 108 (3.8) | 71 (4.4) | 234 (4.3) | 118 (5.6) | 24 (5.1) | 8 (6.6) | 0.037 | 0.057 |
| Ever hysterectomy (%) | 74 (2.6) | 61 (3.8) | 246 (4.5) | 109 (5.2) | 26 (5.6) | 8 (6.6) | <0.001 | 0.083 |
| Ever bilateral oophorectomy (%) | 50 (1.8) | 34 (2.1) | 95 (1.7) | 56 (2.7) | 6 (1.3) | 2 (1.7) | 0.104 | 0.041 |
| Ever hormone therapy (%) | 572 (20.2) | 400 (25.1) | 1585 (28.9) | 644 (30.8) | 143 (30.6) | 28 (23.1) | <0.001 | 0.121 |
| Menopause (%) | 1417 (50.2) | 883 (55.3) | 3383 (61.7) | 1346 (64.4) | 318 (68.1) | 82 (67.8) | <0.001 | 0.178 |
| CHD (%) | 12 (0.4) | 19 (1.2) | 61 (1.1) | 26 (1.2) | 11 (2.4) | 3 (2.5) | 0.001 | 0.079 |
| Hypertension (%) | 377 (13.3) | 245 (15.4) | 808 (14.7) | 343 (16.4) | 92 (19.7) | 23 (19.0) | 0.002 | 0.082 |
| CKD (%) | 17 (0.6) | 9 (0.6) | 27 (0.5) | 9 (0.4) | 4 (0.9) | 1 (0.8) | 0.852 | 0.026 |
| T1DM (%) | 4 (0.1) | 0 (0.0) | 12 (0.2) | 3 (0.1) | 1 (0.2) | 0 (0.0) | 0.528 | 0.037 |
| T2DM (%) | 24 (0.8) | 11 (0.7) | 43 (0.8) | 11 (0.5) | 1 (0.2) | 3 (2.5) | 0.121 | 0.079 |
| HF (%) | 0 (0.0) | 1 (0.1) | 0 (0.0) | 0 (0.0) | 1 (0.2) | 0 (0.0) | 0.009 | 0.030 |
| Stroke (%) | 1 (0.0) | 0 (0.0) | 4 (0.1) | 1 (0.0) | 1 (0.2) | 0 (0.0) | 0.610 | 0.033 |
| Atherosclerosis (%) | 0 (0.0) | 1 (0.1) | 0 (0.0) | 0 (0.0) | 0 (0.0) | 0 (0.0) | 0.230 | 0.012 |

**Supplementary table 8. Weighted baseline characteristics of participants measured bone density**

|  | Parity | | | | | | *P* | *SMD* |
| --- | --- | --- | --- | --- | --- | --- | --- | --- |
|  | 0 | 1 | 2 | 3 | 4 | ≥5 |  |  |
| n | 12625.5 | 12582.98 | 12580.05 | 12521.03 | 12071.65 | 4926.86 |  |  |
| BMD (Neck of Femur) | 0.88 (0.13) | 0.87 (0.13) | 0.88 (0.13) | 0.88 (0.13) | 0.87 (0.13) | 0.87 (0.12) | 0.250 | 0.045 |
| BMD (Spine) | 1.01 (0.15) | 1.00 (0.15) | 1.01 (0.15) | 1.00 (0.15) | 1.00 (0.14) | 1.01 (0.14) | 0.818 | 0.020 |
| Age | 62.65 (7.55) | 62.66 (7.55) | 62.71 (7.82) | 62.76 (7.83) | 62.89 (7.88) | 64.36 (7.50) | 0.301 | 0.082 |
| BMI (mean (SD)) | 25.83 (4.64) | 25.85 (4.40) | 25.73 (4.24) | 25.80 (4.37) | 25.78 (4.36) | 26.75 (4.32) | 0.176 | 0.082 |
| BMI (%) |  |  |  |  |  |  | 0.406 | 0.090 |
| Normal | 6307.2 (50.0) | 6229.2 (49.5) | 6240.5 (49.6) | 6199.1 (49.5) | 5981.8 (49.6) | 1986.0 (40.3) |  |  |
| Underweight | 96.8 (0.8) | 95.9 (0.8) | 106.5 (0.8) | 96.5 (0.8) | 48.7 (0.4) | 54.0 (1.1) |  |  |
| Overweight | 4298.9 (34.0) | 4377.3 (34.8) | 4357.8 (34.6) | 4354.7 (34.8) | 4174.6 (34.6) | 1830.3 (37.1) |  |  |
| Obesity | 1922.6 (15.2) | 1880.6 (14.9) | 1875.2 (14.9) | 1870.7 (14.9) | 1866.5 (15.5) | 1056.5 (21.4) |  |  |
| Ethnicity (%) |  |  |  |  |  |  | 0.997 | 0.045 |
| White | 12210.5 (96.7) | 12156.5 (96.6) | 12152.5 (96.6) | 12103.6 (96.7) | 11762.8 (97.4) | 4791.1 (97.2) |  |  |
| Asian | 144.8 (1.1) | 168.7 (1.3) | 159.5 (1.3) | 151.8 (1.2) | 75.8 (0.6) | 46.2 (0.9) |  |  |
| Black | 97.9 (0.8) | 99.2 (0.8) | 107.2 (0.9) | 96.1 (0.8) | 99.0 (0.8) | 41.8 (0.8) |  |  |
| Mixed | 81.4 (0.6) | 78.0 (0.6) | 78.6 (0.6) | 87.8 (0.7) | 48.1 (0.4) | 31.0 (0.6) |  |  |
| Other | 91.0 (0.7) | 80.6 (0.6) | 82.3 (0.7) | 81.8 (0.7) | 86.1 (0.7) | 16.8 (0.3) |  |  |
| College or University | 6118.7 (48.5) | 6082.4 (48.3) | 6126.4 (48.7) | 6095.8 (48.7) | 5661.4 (46.9) | 2163.5 (43.9) | 0.648 | 0.039 |
| Smoking (%) |  |  |  |  |  |  | 0.909 | 0.044 |
| Never | 8053.7 (63.8) | 8087.1 (64.3) | 8012.4 (63.7) | 8038.1 (64.2) | 7757.0 (64.3) | 3105.8 (63.0) |  |  |
| Occasionally | 1631.4 (12.9) | 1689.9 (13.4) | 1704.1 (13.5) | 1692.5 (13.5) | 1569.3 (13.0) | 816.5 (16.6) |  |  |
| Daily or almost daily | 2940.4 (23.3) | 2806.0 (22.3) | 2863.6 (22.8) | 2790.5 (22.3) | 2745.3 (22.7) | 1004.5 (20.4) |  |  |
| Drinking (%) |  |  |  |  |  |  | 0.027 | 0.147 |
| Never | 657.8 (5.2) | 636.5 (5.1) | 627.6 (5.0) | 628.1 (5.0) | 623.3 (5.2) | 466.6 (9.5) |  |  |
| Special occasions only | 1321.2 (10.5) | 1295.4 (10.3) | 1280.0 (10.2) | 1250.2 (10.0) | 1251.7 (10.4) | 901.0 (18.3) |  |  |
| One to three times a month | 1627.5 (12.9) | 1586.9 (12.6) | 1595.4 (12.7) | 1626.5 (13.0) | 1424.1 (11.8) | 833.2 (16.9) |  |  |
| Once or twice a week | 3288.0 (26.0) | 3298.1 (26.2) | 3310.2 (26.3) | 3287.3 (26.3) | 3141.5 (26.0) | 937.4 (19.0) |  |  |
| Three or four times a week | 3287.4 (26.0) | 3395.7 (27.0) | 3358.1 (26.7) | 3351.9 (26.8) | 3431.1 (28.4) | 1187.7 (24.1) |  |  |
| Daily or almost daily | 2443.6 (19.4) | 2370.4 (18.8) | 2408.7 (19.1) | 2376.9 (19.0) | 2199.9 (18.2) | 601.0 (12.2) |  |  |
| Healthy diet (%) | 8283.8 (65.6) | 8196.7 (65.1) | 8254.5 (65.6) | 8196.3 (65.5) | 7555.0 (62.6) | 3178.4 (64.5) | 0.720 | 0.026 |
| IPAQ (%) |  |  |  |  |  |  | 0.698 | 0.061 |
| Low | 2196.1 (17.4) | 2176.4 (17.3) | 2180.4 (17.3) | 2129.7 (17.0) | 2233.3 (18.5) | 955.7 (19.4) |  |  |
| Moderate | 5563.7 (44.1) | 5494.3 (43.7) | 5508.5 (43.8) | 5472.2 (43.7) | 5209.6 (43.2) | 1802.6 (36.6) |  |  |
| High | 4865.7 (38.5) | 4912.3 (39.0) | 4891.2 (38.9) | 4919.2 (39.3) | 4628.7 (38.3) | 2168.5 (44.0) |  |  |
| Area (%) |  |  |  |  |  |  | 0.990 | 0.031 |
| Urban | 10618.7 (84.1) | 10605.0 (84.3) | 10616.2 (84.4) | 10524.1 (84.1) | 10252.5 (84.9) | 4167.1 (84.6) |  |  |
| Town | 952.6 (7.5) | 904.5 (7.2) | 909.2 (7.2) | 912.4 (7.3) | 852.4 (7.1) | 294.1 (6.0) |  |  |
| Village | 1054.2 (8.3) | 1073.5 (8.5) | 1054.6 (8.4) | 1084.6 (8.7) | 966.7 (8.0) | 465.7 (9.5) |  |  |
| Employment (%) |  |  |  |  |  |  | 0.253 | 0.101 |
| Unemployed | 951.3 (7.5) | 839.9 (6.7) | 840.1 (6.7) | 852.8 (6.8) | 866.0 (7.2) | 639.6 (13.0) |  |  |
| Full- or part-time student | 8828.0 (69.9) | 8897.8 (70.7) | 8877.6 (70.6) | 8829.4 (70.5) | 8476.1 (70.2) | 3331.3 (67.6) |  |  |
| Doing unpaid or voluntary work | 94.6 (0.7) | 98.1 (0.8) | 90.6 (0.7) | 87.2 (0.7) | 100.6 (0.8) | 99.1 (2.0) |  |  |
| Retired | 57.2 (0.5) | 58.7 (0.5) | 56.2 (0.4) | 57.7 (0.5) | 46.2 (0.4) | 0.0 (0.0) |  |  |
| In paid employment or self-employed | 2694.4 (21.3) | 2688.5 (21.4) | 2715.5 (21.6) | 2693.9 (21.5) | 2582.7 (21.4) | 856.9 (17.4) |  |  |
| Townsend index | -1.85 (2.77) | -1.79 (2.74) | -1.80 (2.75) | -1.84 (2.65) | -1.83 (2.70) | -1.21 (3.05) | 0.339 | 0.078 |
| Menarche age (%) |  |  |  |  |  |  | 0.984 | 0.032 |
| ≤11 | 2258.1 (17.9) | 2206.8 (17.5) | 2188.9 (17.4) | 2226.3 (17.8) | 2083.3 (17.3) | 905.7 (18.4) |  |  |
| 12-15 | 9751.3 (77.2) | 9682.1 (76.9) | 9732.8 (77.4) | 9653.4 (77.1) | 9382.5 (77.7) | 3703.1 (75.2) |  |  |
| ≥16 | 616.0 (4.9) | 694.0 (5.5) | 658.4 (5.2) | 641.3 (5.1) | 605.8 (5.0) | 318.0 (6.5) |  |  |
| S_PRS_EBMDT (mean (SD)) | 0.01 (0.97) | 0.00 (1.00) | 0.00 (0.97) | 0.00 (0.96) | 0.04 (0.98) | 0.02 (0.97) | 0.989 | 0.017 |
| Weak grip strength (%) | 568.6 (4.5) | 580.5 (4.6) | 556.5 (4.4) | 560.3 (4.5) | 611.5 (5.1) | 311.6 (6.3) | 0.699 | 0.034 |

**Supplementary Figure 1.** Balance of variables (SMD) across parity before and after IPTW adjustment in the main analysis. Weights were truncated at the 99th percentile to reduce the influence of extreme weights.

**Supplementary Figure 2.** Balance of variables (SMD) across parity before and after IPTW adjustment in the main analysis in participants with measured bone density. Weights were truncated at the 99th percentile to reduce the influence of extreme weights.
